# Microevolution and Gain or Loss of Mobile Genetic Elements of Outbreak-Related *Listeria monocytogenes* in Food Processing Environments Identified by Whole Genome Sequencing Analysis

**DOI:** 10.3389/fmicb.2020.00866

**Published:** 2020-05-29

**Authors:** Helen Yang, Maria Hoffmann, Marc W. Allard, Eric W. Brown, Yi Chen

**Affiliations:** Center for Food Safety and Applied Nutrition, Food and Drug Administration, College Park, MD, United States

**Keywords:** long-read sequencing, *Listeria monocytogenes*, prophage, outbreak, mobile genetic elements

## Abstract

Whole genome sequencing (WGS) analyses have been instrumental in traceback investigations of *Listeria monocytogenes* (*Lm*). To demonstrate how long-read sequencing analysis can capture and describe relationships among isolates from clinical, food, and environmental sources, we analyzed 366 long-read- and shotgun-sequenced isolates from 16 *Lm* outbreak strains associated with cantaloupe, leafy green, stone fruit, caramel apple, mung bean sprout, multiple cheese products, multiple ice cream products, and their production environments. The analyses demonstrated that outbreak strains could be distributed in different areas and zones of food production environments through persistent or repeated contamination. Multi-strain and multi-clone contamination were common. Further, WGS could differentiate among isolates collected at different time points or from different production lines in the same facility, revealing microevolution events in processing environments. Our comparison between complete and shotgun genomes showed that isolates of the same outbreak strain diversified mostly by gain/loss of plasmids and chromosome-borne prophages that constitute 2 to 5% of the chromosome. In contrast, other genes missing in the shotgun genomes were randomly scattered, constituting ~0.5% of the chromosome. Among different outbreak strains of the same CC, most gene-scale differences were due to gain/loss of mobile genetic elements, such as plasmids, chromosome-borne prophages, a Tn916 like transposon, and *Listeria* Genomic Island 2. The nucleotide variations in the same prophage and the same plasmid shared among isolates of the same outbreak strain were limited, which enabled different WGS tools to unambiguously cluster isolates of the same outbreak strain. In some outbreak strains, correlation between prophage gain/loss and single nucleotide polymorphism (SNP) accumulations in the genome backbone were observed.

## Introduction

*Listeria monocytogenes* (*Lm*) is a foodborne pathogen that can persist in or become repeatedly introduced to food-processing facilities (Orsi et al., 2008), causing food contamination and subsequent listeriosis, a potentially fatal illness. Contamination of *Lm* in meat and poultry, dairy, produce, and seafood processing environments have been reported (Orsi et al., 2008; Leong et al., 2015; Chen et al., 2017b; Tan et al., 2019). To characterize areas in the producing environment according to the potential for product contamination, the U.S. Food and Drug Administration (FDA) has defined a zone system. Specifically, Zone 1 represents food-contact surfaces (FCS), Zone 2 represents non-FCS very close to food and FCS, Zone 3 represents non-FCS within or near processing areas that could lead to contamination of Zones 1 and 2, and Zone 4 represents non-FCS outside the processing areas (U.S. Food and Drug Administration, 2017). Whole genome sequencing (WGS) has been valuable for identifying and describing genomic variations in prophages, other mobile genomic islands, and core genomes of *Lm* during both long-term and short-term evolution (Kuenne et al., 2013). For example, during an outbreak which represents a short-term evolution scenario. *Lm* isolates can share the same prophage with limited diversity (Chen et al., 2016b, 2017a); in some cases, *Lm* could gain or lose prophages over that same short period of time, causing changes in pulsed-field gel electrophoresis (PFGE) banding patterns among isolates associated with one outbreak (Gilmour et al., 2010; Chen et al., 2017b; Li et al., 2017). In long-term evolution scenarios, especially those involving multiple serotypes and genetic lineages, the majority of gene-scale differences (i.e., presence/absence of genes) in *Lm* occurred in mobile genetic elements (MGEs), such as hypervariable hotspots, prophages, transposons, and mobile genomic islands (Kuenne et al., 2013). Recombination in prophages or other MGEs could generate an abundance of nucleotide variations (Orsi et al., 2008). Thus, MGEs could offer valuable information on the persistence and evolution of *Lm*. To study its biodiversity, previous studies have classified *Lm* into genetic lineages, serotypes, and clones (Ragon et al., 2008). A nomenclature system to define *Lm* clones is clonal complex (CC), which includes a group of multilocus sequence typing-defined sequence types (STs) in which any ST differs from at least one other ST by no more than one allele (Ragon et al., 2008). Under this definition, a singleton includes a group of isolates that differed from any existing STs of the species by at least 2 alleles (Ragon et al., 2008).

WGS enables precision in outbreak investigation and source tracking of foodborne pathogens (Lüth et al., 2018). Most of these efforts employ shotgun sequencing, which breaks a genome into short DNA fragments for sequencing. Bioinformatics tools are then used to assemble short DNA fragments into longer contigs, and the final assembled genome contains multiple contigs in random orders. One potential issue of shotgun sequencing is that a large MGE, such as a prophage, contains repetitive sequences (Dorscht et al., 2009) and may not be assembled into the same contig, making identification of the complete MGE difficult. Long-read sequencing, which can close the entire genome or generate very long contigs, offers the solution to identify complete sequences of large MGEs. Currently, long-read sequencing is relatively costly due to the lower throughput and the need for expensive capital equipment. In addition, error rates of long-read sequencing appear to be slightly higher than shotgun sequencing. These disadvantages are expected to improve with the evolution of sequencing technologies and development of advanced platforms (Lüth et al., 2018; Gonzalez-Escalona et al., 2019).

WGS analytic tools target different regions of genomes such as the entire genome (Davis et al., 2015), core genome (Nielsen et al., 2017; Jagadeesan et al., 2019), coding regions of the entire genome (Jackson et al., 2016), and coding regions of the core genome (Chen et al., 2016b). When analyzing the microevolution events of isolates in a food production environment, maximum resolution may be needed to differentiate various isolates of the same outbreak strain. For example, in a hospital-acquired listeriosis outbreak associated with milkshake contaminated with a CC101 strain, 2 SNPs in the entire genome were critical in identifying a clade of isolates that persisted in a milkshake machine for 1 year and caused illnesses 1 year apart; those 2 SNPs separated the persistent isolates from other isolates within the outbreak cluster (Li et al., 2017). For another example, variations due to recombination of prophage regions and possible prophage replacement differentiated CC11 isolates that were isolated from the same meat/poultry facility 12 years apart (Orsi et al., 2008; Chen et al., 2016b). In order to maximize the resolution of core genome multilocus sequence typing (MLST), a core genome could be defined based on a specific collection of isolates, instead of the entire species. For *Lm*, we could define a core genome for a genetic lineage, a serotype, or a CC (Chen et al., 2016b; Li et al., 2017). When a core genome is defined from isolates of the same outbreak strain, the resulting core genome MLST scheme essentially targets the entire genome of the outbreak strain, including MGEs.

Here, we employed complete genomes and strain-specific core genome MLST to analyze *Lm* associated with recent listeriosis outbreaks and to determine the persistence/repeated contamination, transmission, and microevolution of *Lm* in these outbreaks. We also used the complete genomes to study the gene-scale differences among isolates associated with each outbreak strain.

## Materials and Methods

### Whole Genome Sequences

From FDA GenomeTrakr (https://www.ncbi.nlm.nih.gov/bioproject/541969), we selected the sequences from isolates associated with select listeriosis outbreaks after 2011. In addition, we selected available isolates collected as part of the outbreak investigations but not associated with any clinical cases, isolates collected as part of surveillance samplings prior to and/or after the outbreak investigation from implicated facilities, and isolates initially suspected to be associated with outbreaks (Table 1, Table S1). Several polyclonal outbreaks had multiple outbreak strains. The isolates associated with each outbreak strain were previously determined by epidemiological investigations and WGS analyses (McCollum et al., 2013; Choi et al., 2014; Centers for Disease Control and Prevention, 2015b,c, 2017; Jackson et al., 2015; Self et al., 2016; Angelo et al., 2017; Chen et al., 2017a,b). We chose all available environmental isolates; if a relatively large number of isolates was available, we chose a portion of the food and clinical isolates that best represented collection dates, diversity, presence/absence of MGEs, and evolutionary history discussed in this study. In addition to shotgun genomes, we also obtained available complete genomes of both chromosomes and plasmids from these isolates, which were sequenced by PacBio technology. If an isolate was subjected to both long-read and shotgun sequencing, we obtained both genomes. In case a complete genome was not available for an outbreak strain, we selected an unrelated complete genome of the same CC. We used CLC Genomics Workbench 11 (Qiagen, Hilden, Germany) to assemble the shotgun genomes using default adapter trimming and *de novo* assembly settings. Sequences chosen met the following quality criteria: sequencing coverage of ≥30x and sequences containing ≥95% of the 1,827 previously identified core genes of the entire population of *Lm* (Chen et al., 2016b). For each analysis, outgroups were not related to the outbreak but exhibited either the same PFGE profile or the same CC profile as the outbreak strain, and their WGS sequences were downloaded from GenomeTrakr.

**Table 1:** *Lm* analyzed in the present study.

**Subsection in analysis of individual strains^**a**^**	**Food vehicle**	**Geographic range**	**Year(s)**	**ST^**b**^, CC^**b**^ and serogroup**	**Prophage presence in at least one, all isolates**	**Plasmid presence in at least one, all isolates**	**Source^**c**^**	**Accession of complete genome^**d**^**
1	Mung bean sprout	Multistate	2014	ST554, CC554, IVb-v1	Yes, No^e^	No, No	C, F, E	CP043177.2
2	Hispanic-style cheese	Maryland	2013	ST5, CC5, IIb	Yes, Yes	Yes, No	C, F, E	CP014250.2
3	Artisan cheese	Multistate	2013	ST6, CC6, IVb	No, No	No, No	C, F, E	CP007600.2
4	Soft cheese	Multistate	2010-15	ST6, CC6, IVb	Yes, No	Yes, No	C, E	CP044432.2
5	Cantaloupe (strain #1)	Multistate	2011	ST5, CC5, IIb	No, No	No, No	C, F, E	NZ_CP007686.1
6	Cantaloupe (strain #2)	Multistate	2011	ST7, CC7, IIa	Yes, Yes	No, No	C, E	NZ_CP007685.1
	Cantaloupe (strain #3)	Multistate	2011	ST561, CC7, IIa	Yes, Yes	No, No	C, E	NZ_CP007684.1
7	Caramel apple (strain #1)	Multistate	2014-15	ST1, CC1, IVb	Yes, Yes	No, No	C, F, E	CP006596.2
	Soft cheese	Multistate	2017	ST1, CC1, IVb	Yes, Yes	No, No	C, F	CP006596.2
8	Hispanic-style cheese	Multistate	2014	CC2, IVb	Yes, No	Yes, Yes	C, F, E	CP006046.4
9	Caramel apple (strain #2)	Multistate	2014-15	ST382, IVb-v1	No, No	No, No	C, F, E	CP012021.2
	Stone fruit	Multistate	2014	ST382, IVb-v1	No, No	No, No	C, F, E	CP012021.2
	Leafy green	Multistate	2015-16	ST382, IVb-v1	No, No	No, No	C, F, E	CP012021.2
10	Ice cream (strain #1)	Multistate	2010-15	ST5, CC5, IIb	Yes, No	Yes, No	C, F, E	CP016213.2
	Ice cream (strain #2)	Multistate	2010-15	ST5, CC5, IIb	Yes, No	Yes, No	C, F, E	CP016213.2
	Ice cream	Florida	2017	ST5, CC5, IIb	Yes, No	Yes, No	C, E	CP016213.2
11	Stone fruit (non-outbreak)	California	2014	ST5, CC5, IIb	Yes, No	Yes, No	F, E	CP014252.2

a The number corresponds to the subsection in the Analysis of Individual Strains in the Results and Discussion section. Each subsection describes a strain-specific cgMLST analysis. The number also corresponds to the figure numbers and numbers in Tables S1–S3.

b ST, sequence type; CC, clonal complex.

c C, clinical; F, food; E, environmental.

d Complete genomes used to analyze each outbreak strain. The complete genome is not necessarily from an isolate associated with the outbreak.

e *Yes indicates presence and no indicates absence. The first yes indicates presence in at least one isolate, and the second yes indicates presence in all isolates*.

*In-silico* ibMLST and molecular serogroup identification were performed on the isolates using the MLST and molecular serogrouping tools in the SeqSphere^+^ software (Ridom GmbH). CCs were then assigned using the definition given by Ragon et al. (2008) and profiles curated in the Pasteur MLST database (http://bigsdb.pasteur.fr/listeria/listeria.html; Moura et al., 2016).

### Comparison Between Long-Read- and Shotgun-Sequenced Isolates of the Same Outbreak Strain by BLAST and Strain-Specific Core Genome MLST

Among isolates of the same outbreak strain, we used the gene-by-gene BLAST function built in SeqSphere^+^ (Ridom GmbH, Germany) to determine whether any genes of the complete genome were present in the shotgun genomes (i.e., gene-scale differences). If a complete genome was not available for any isolate of an outbreak strain, we used the unrelated complete genome of the same CC. Subsequently, we used the cgMLST Target Definer (version 3.1.0) function of SeqSphere^+^ (Ridom GmbH, Germany) with default parameters as described in Ruppitsch et al. (2015) to define multiple strain-specific core genome MLST schemes from complete chromosomes. This software begins with one designated genome, named as the seed genome, and uses a given set of genomes as BLAST queries to identify shared protein-coding genes. Subsequently the software filters out the following genes: all genes ≤ 50 base pairs (bp), genes containing no start codon or stop codon, those containing premature stop codons and those containing fragments occurring in multiple copies. In the case of overlapping genes, the longer gene was selected as the core gene (Ruppitsch et al., 2015). In this study, we defined strain-specific core genome MLST schemes using only the seed genome without any query genomes. We created separate strain-specific cgMLST schemes for each outbreak strain, except that if several outbreak strains belonged to the same CC, but a complete chromosome was available for only one strain, we defined one strain-specific cgMLST using that complete chromosome and analyzed multiple outbreak strains together.

Each gene was extracted from an isolate using the default parameters in SeqSphere^+^ (Ridom GmbH) as described in Ruppitsch et al. (2015) and compared with the cgMLST core genome by BLAST. The presence of core genes in each genome was thus determined. The allele for each gene was automatically assigned by SeqSphere^+^ (Ridom GmbH), and the combination of alleles of all core genes formed the allelic profile for an isolate. Allelic profiles of multiple isolates were then used to generate neighbor-joining (NJ) trees with the parameter “pairwise ignore missing values” during distance calculations. When presenting the phylogenetic trees, we included all environmental isolates and a portion of representative clinical and food isolates to allow better visualization of environmental isolates in the trees. All the trees were rooted at midpoint. For each analysis, we determined the maximum number of allelic differences in pairwise comparisons and performed single-linkage analysis using the minimum-spanning tree tool in SeqSphere^+^ to determine the maximum number of allelic differences between any two neighboring isolates.

### Analysis of Chromosome-Borne Prophages, Other Chromosome-Borne MGEs, and Plasmids

For complete genomes, we performed PHASTER (Arndt et al., 2016) to predict the chromosome-borne prophages, using the NCBI accession numbers as the input. We did not investigate plasmid-borne prophages. For our analyses, we only considered prophages identified to be “complete” and “questionable” as positive identifications by PHASTER. PHASTER predicted a 10.7 Kb prophage as “questionable” in all *Lm* genomes surveyed here. This prophage was likely a previously described monocin, a defective or satellite prophage, in F2365 (Nelson et al., 2004) and thus, it was not discussed in this study. We used three methods to identify prophages in shotgun-sequenced isolates. First, we performed PHASTER analysis directly on shotgun genomes. Second, we identified gene-scale differences among the long-read- and shotgun-sequenced isolates of the same outbreak strain using gene-by-gene BLAST built in SeqSphere^+^ and determined if any genes of a prophage predicted by PHASTER from the complete genome were present in the shotgun-sequenced isolates. Third, we used BLAST to determine if a shotgun-sequenced isolate contained a prophage predicted by PHASTER from another isolate of the same outbreak strain. If a complete genome was available for an isolate, we used the prophage(s) predicted from that complete genome as the BLAST query. If no complete genome was available, we used the prophage(s) predicted from a shotgun genome as the BLAST query. We viewed a genome that contained ≥90% (i.e., query coverage) of a prophage with ≥98% sequence identity as containing that prophage; if a genome contained ≤ 40% of a prophage, we viewed that genome as missing that prophage. We chose 40% because PHASTER-predicted prophage ends may be slightly different from the actual prophage insertion sites, as discussed below. For the same reason, when we used BLAST to directly compare two PHASTER-predicted prophages, we used ≥70% query coverage (QC) and >98% sequence identity (SI) to determine whether the two prophages were the same.

We also determined the presence of plasmid(s) in shotgun genomes. We used the contigs of each shotgun genome as BLAST queries to compare with complete sequences of 52 *Listeria* plasmids deposited in the GenBank as of November 10, 2019, and we searched for the *repA* gene in all shotgun genomes. We viewed a contig as a plasmid contig if the QC was ≥60% and SI was ≥70%. We viewed *repA* as present in a shotgun genome if the BLAST had ≥60% QC and ≥70% SI. We chose 60% as the cut-off for QC in case a novel plasmid only partially aligned with published plasmids. Around 90% of the published plasmids were ≥10 Kb; in addition, plasmids and chromosomes shared homologous regions (Kuenne et al., 2010), thus, we determined that a shotgun genome contained a plasmid if the combined length of plasmid contigs exceeded 10 Kb and if *repA* was present in a plasmid contig. If the combined length of plasmid contigs was less than 10 Kb, we viewed the determination of plasmid presence as inconclusive. When PacBio long-read sequencing of an outbreak isolate identified a plasmid, we used this plasmid as the BLAST query to compare with the shotgun genomes from the same outbreak. We viewed a shotgun-sequenced isolate containing the plasmid with QC ≥80% and SI ≥98%. We chose 80% as the cut-off in case shotgun sequencing did not provide sufficient coverage of the entire plasmid.

When comparison between complete genomes and shotgun genomes revealed the gain/loss of an MGE other than prophages or plasmids, this MGE was identified by examining its protein functions. If needed, we compared the genome using Mauve (Darling et al., 2004) or Artemis Comparison Tool (ACT) (Carver et al., 2005) to illustrate the gain/loss/recombination of MGEs.

### Center for Food Safety and Applied Nutrition (CFSAN) SNP Pipeline Analysis and Bayesian Evolutionary Analysis by Sampling Trees (BEAST)

We performed FDA CFSAN SNP Pipeline analysis (v0.6.0) on outbreak strains that had not been previously analyzed by such pipeline, according to the previously described protocol (Davis et al., 2015; Chen et al., 2017b). Briefly, raw reads from each shotgun genome were mapped to the reference genome with Bowtie2 version 2.2.2 (Langmead et al., 2009). The BAM file was sorted using Samtools version 0.1.19 (Li et al., 2009), and a pileup file for each genome was produced. These files were then processed using VarScan2 version 2.3.9 to identify high quality variant sites (Koboldt et al., 2009). A Python script in the pipeline was used to parse the .vcf files and construct an initial SNP matrix. A filter was applied to exclude variant sites in high-density variant regions (i.e., containing ≥3 variant sites in ≤ 1,000 bp of any one genome) since they may be the result of recombination, or low-quality sequencing/mapping often occurring in repetitive regions (Chen et al., 2017b,c). The complete chromosome used for each strain-specific cgMLST scheme was used as the reference genome for each CFSAN SNP Pipeline analysis. We performed the SNP Pipeline analysis twice for each outbreak strain, one with outgroup to demonstrate how SNP analysis separated the outbreak isolates from the outgroup, and the other without outgroup to precisely determine the pairwise SNP distances among isolates. GARLI was used to construct maximum likelihood trees based on SNPs among outbreak isolates and outgroup.

In 3 outbreaks, isolates were isolated from more than 3 years. To further understand the evolution of isolates (Table S1) associated with these outbreaks, we took advantage of the heterochronous sampling of individuals and estimated divergence dates among these isolates using BEAST v2.6.1 (Bouckaert et al., 2019). The Hasegawa-Kishino-Yano (HKY) model of nucleotide substitution was used, gamma category was set to 4, and kappa initial estimate was set to 4.0. Strict, relaxed lognormal, and relaxed exponential models were explored along with tree priors assuming a coalescent constant population, coalescent exponential population, and coalescent Bayesian skyline population. Monte Carlo Markov Chain (MCMC) length was set to 100 million for each run and tracelog and treelog were recorded every 2,000 runs. Tracer v.1.7.1 was used to retrieve results and confirm that the effective sample size (ESS) values were above 200. Nested sampling (Russel et al., 2019) with 200,000 chain length and 32 particle counts was performed and marginal likelihoods were compared to determine the best supported model and priors. After that, five independent runs were performed; the results were combined using LogCombiner v2.6.1 (Bouckaert et al., 2019). The combined trees were then sampled using TreeAnnotator v2.6.1 (Bouckaert et al., 2019) with a 10% burn-in to obtain a tree with maximum clade credibility and with node heights being “common ancestor heights.” The current date was the most recent isolation date among any isolates of the outbreak strain.

## Results and Discussion

### Analysis of Individual Outbreak Strains

#### 1. Multistate, Mung Bean Sprouts, 2014 (Centers for Disease Control and Prevention, 2015c) (ST554, CC554, Serogroup IVb-v1)

This sprout-associated outbreak was the first outbreak known to be caused by a strain of CC554. Analyses have shown that CC554 belongs to the serogroup IVb variant 1 (IVb-v1), also known as the serotype 4b variant. By traditional antiserum-based typing, isolates of this serogroup are serotype 4b, a part of molecular serogroup IVb; however, these isolates also contain genetic markers for serogroups IVb, IIa, and IIc (Lee et al., 2012).

We used the complete genome of an outbreak isolate from sprouts (FDA00008248, NCBI Accession: CP043177.2; 2,873 protein coding regions) and determined that shotgun genomes of outbreak isolates, including the shotgun genome of FDA00008248, contained 96.7–99.8% of the genes in the complete FDA00008248 genome. Among them, 16 shotgun genomes contained 99.4–99.8% of complete set of FDA00008248 genes. The genes missing in these 16 shotgun genomes were randomly scattered across the complete genome, and we could not determine if these genes were genuinely missing in the isolates or if these genes were missing due to artifacts of shotgun sequencing. Shotgun genomes of the other 25 isolates contained 96.7–97.2% of the genes in the complete FDA00008248 genome. The difference was largely due to these 25 shotgun genomes missing a 47 Kb region (FDA00008248 genome positions: 1688065 to 1735327, 72 genes, 2.5% of the complete gene set), which was a major part of the prophage (54 Kb) predicted from the complete FDA00008248 genome (Tables S1, S2). It is unlikely that 72 genes in an entire 47 Kb region were not covered by shotgun sequencing, so we considered these genes to be genuinely missing in those isolates. BLAST comparison between the predicted FDA00008248 prophage and shotgun-sequenced isolates also showed this prophage was present in the 16 isolates [100% query coverage (QC) and >99.9% sequence identity (SI)], but not in the other 25 isolates (QC <11%). PHASTER analysis performed directly on shotgun genomes predicted a 48 Kb prophage from 16 isolates (Table S1), which corresponded to a major part of the FDA00008248 prophage predicted from the complete genome. It is possible that the prophage ends predicted by PHASTER are slightly different from the actual prophage insertion sites (Chen et al., 2017b); this possibility applies to all analyses and we do not mention this again from this point on. The loss of the FDA00008248 prophage would result in a change of the ~890 Kb *Asc*I-pulsed field gel electrophoresis (PFGE) fragment (between *Asc*I restriction sites at genome positions ~1208452 and ~2098242) to ~842 Kb. DNA fragments of such large size could not be resolved by PFGE, explaining why the *Asc*I-PFGE banding pattern was identical among all isolates (U.S. CDC PulseNet PFGE pattern ID, GX6A16.0319). Long-read sequencing did not identify any plasmid in FDA00008248, and our analysis of all shotgun genomes, including that of FDA00008248, did not reveal any plasmid.

To gain more insights on the differences among different strains of the same CC, we compared FDA00008248 with the shotgun-sequenced unrelated outgroup FSIS1503333, which exhibited the outbreak-associated PFGE profile and ST, and found that the major gene-scale differences were due to FSIS1503333 not having the FDA00008248 prophage.

**Figure 1:**
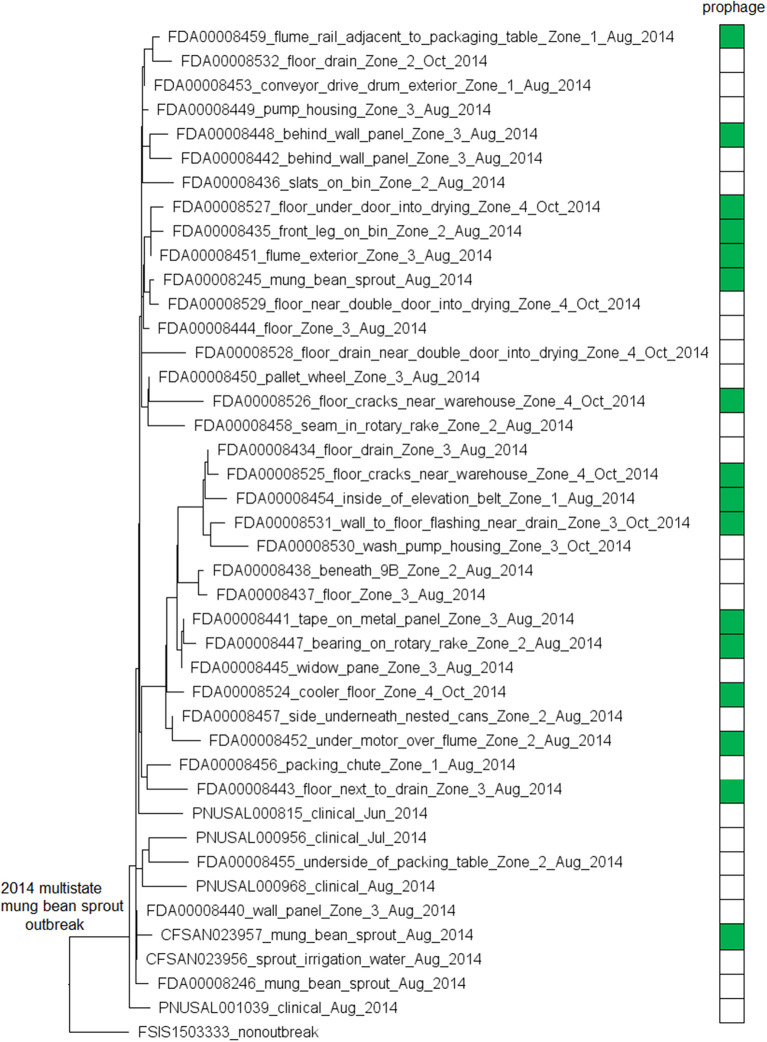
Neighbor joining (NJ) tree of selected available WGS data for the 2014 multistate mung bean sprout outbreak strain. All environmental isolates and a portion of representative food and clinical isolates are included in the tree. Environmental isolate ID is followed by facility location, zone information (when available), and isolation date. Clinical and food isolate ID is followed by the source and isolation date. A CC554 environmental isolate (FSIS1503333) that shared the same PFGE as the outbreak isolates serves as the non-outbreak outgroup. Some isolates contained a prophage; the green filled space to the right of the tree indicates the presence of the prophage and the open space indicates the absence of the prophage. Prophage gain/loss did not correlate with phylogenetic clades. FSIS1503333 did not contain the prophage. Isolates did not contain any plasmid.

The environmental isolates were collected in August and October 2014 from separate locations of Zones 1, 2, 3, and 4, such as floors, drains, equipment legs and wheels, wall panels, window panes, processing tables, and equipment, while the clinical isolates were collected between June and August 2014 (Centers for Disease Control and Prevention, 2015c). We used the complete FDA00008248 chromosome as the seed genome without query genomes and filtered out genes not suitable as cgMLST targets to define a core genome MLST containing 2,669 genes. A neighbor-joining (NJ) tree showed a close relationship among all outbreak-associated isolates, which helped indicate that the outbreak strain had spread across multiple zones and areas of the facility. Notably, an irrigation water sample also yielded an outbreak isolate (CFSAN023956, Figure 1). The isolates with or without the prophage did not form a monophyletic clade, indicating that the gain/loss of this prophage did not correlate with the nucleotide variations in the genome backbone. The outbreak isolates differed by ≤ 13 cgMLST alleles in pairwise comparisons and the maximum linkage in the minimum spanning tree was 4 alleles. The prophage contributed to maximal 3 allelic differences by cgMLST (Table S3). Meanwhile, CFSAN SNP Pipeline analysis showed that isolates differed by ≤ 12 SNPs with a maximum linkage of 4 SNPs (Table S3), and the prophage contributed to maximal 4 SNPs. In the resulting SNP-based maximum likelihood tree, the isolates not having the prophage also did not form a clade (Figure S1).

#### 2. Maryland, Hispanic Style Cheese, 2013 (Chen et al., 2017a) (ST5, CC5, Serogroup IIb)

**Figure 2:**
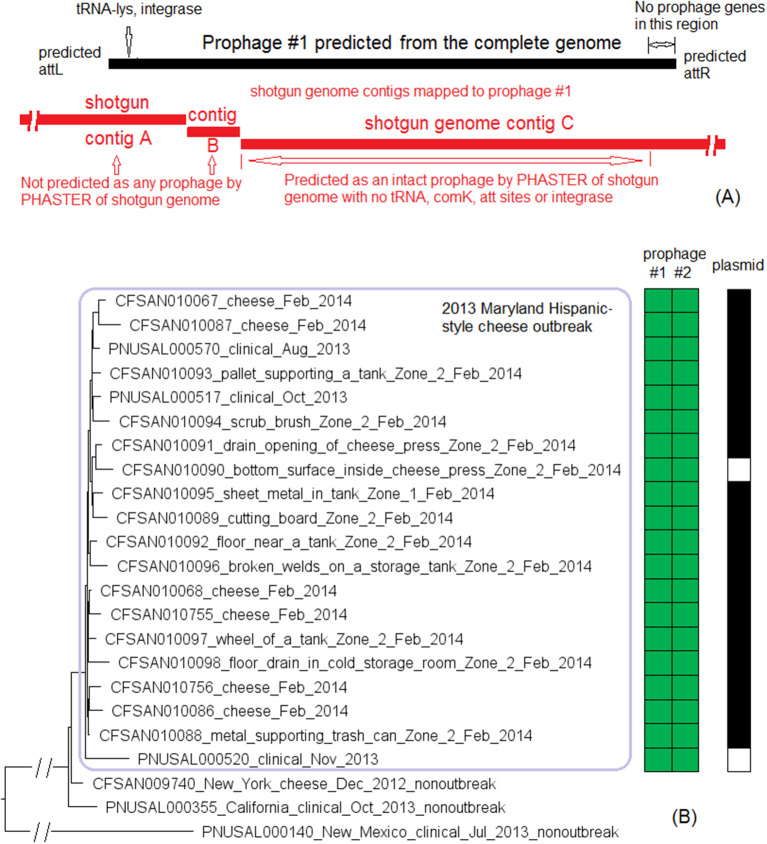
**(A)** Comparison between prophage #1 predicted from the CFSAN010068 complete genome and the PHASTER prediction from the CFSAN010068 shotgun genome. The figure is for illustration purposes and the lengths of genomic contigs or regions are not necessarily proportional to their actual lengths. This prophage was split into 3 contigs in the shotgun genome, arbitrarily named as A, B, C. PHASTER performed directly on shotgun genomes did not predict any prophages in contigs A or B even though they contained phage genes, integrase, and tRNA prophage insertion sites. PHASTER predicted an intact prophage in the longer contig C, which corresponded to 58% of prophage #1. This predicted prophage ended ~3 Kb before the end of the CFSAN010068 prophage #1. This ~3 Kb region did not contain any prophage genes but was predicted as part of CFSAN010068 prophage #1 probably because the attachment site was predicted at the end of the ~3 Kb region. **(B)** NJ tree of selected available WGS data for the 2013 Maryland Hispanic-style cheese outbreak strain. All environmental isolates and a portion of representative food and clinical isolates are included in the tree. Environmental isolate ID is followed by facility location, zone information (when available), and isolation date. Clinical and food isolate ID is followed by the source and isolation date. The outbreak cluster is enclosed in the purple box. Three unrelated isolates serve as the outgroup. The branch lengths of the outgroup PNUSAL000140 were manually reduced to allow better visualization of different clades inside the outbreak cluster. All isolates of this outbreak strain contained two prophages (#1 and #2), and most isolates contained a plasmid. To the right of the tree, green filled spaces indicate the presence of prophages, the black filled space indicates the presence of the plasmid, and the open space indicates the absence of the plasmid.

We used the complete genome of an outbreak isolate from cheese (CFSAN010068, chromosome NCBI Accession: CP014250.2; 2,893 protein-coding regions; plasmid pCFSAN010068_01, NCBI Accession: NZ_CP014251.1, 57 protein-coding regions) and determined that the shotgun genomes of all outbreak isolates contained 99.1–99.6% of genes in the complete CFSAN010068 chromosome. The genes missing in those shotgun genomes were randomly scattered across the complete chromosome, indicating that shotgun-sequenced isolates did not miss any chromosome-borne genomic islands that may be carried within CFSAN010068. This was consistent with our PHASTER and BLAST analyses. PHASTER predicted 2 prophages to be present in the complete CFSAN010068 genome, CFSAN010068 prophage #1 (47 Kb) and CFSAN010068 prophage #2 (45 Kb), containing 59 and 57 genes, respectively (Table S2). The BLAST analysis showed that all shotgun-sequenced isolates contained both prophages (100% QC and >99.9% SI). Indeed, losing either prophage by an isolate would have caused its shotgun genome to miss at least ~2% of genes in the complete CFSAN010068 chromosome. PHASTER performed directly on shotgun genomes of all the outbreak isolates, including the shotgun genome of CFSAN010068, consistently predicted two prophages, 27 and 34 Kb (Tables S1, S4), but these corresponded to only 58 and 76% of CFSAN010068 prophage #1 and CFSAN010068 prophage #2, respectively, with >99.9% SI in BLAST alignments. Closer examination of the PHASTER results led us to believe that the predictions from the complete genome were more accurate. For example, CFSAN010068 prophage #1 predicted from the complete genome was split into 3 fragments found in 3 contigs of a shotgun genome (Figure 2A). The prophage predicted from shotgun genomes corresponded to a large portion of CFSAN010068 prophage #1, but the entire CFSAN010068 prophage #1 could not be directly predicted from any shotgun genome (Table S1, Figure 2A), even though shotgun sequencing of many isolates had >80 × coverage.

The plasmid in CFSAN010068 was found in all shotgun-sequenced isolates except an environmental isolate (CFSAN010090) and a clinical isolate (PNUSAL000520); in addition, nucleotide variations occurred in two genes of the plasmid, RS15075 and RS15140, both of which were IS21 family transposase. This plasmid contained Tn5422 transposon which had *cadA1C1*, a cassette involved in *Lm* resistance to cadmium (Parsons et al., 2017).

The environmental isolates were collected in February 2014 from various Zone 1 and Zone 2 locations, such as the floor, pallet, drains, wheels, and processing equipment, while the cheese isolates were collected in February 2014 and the clinical isolates were collected between August and November 2013 (Centers for Disease Control and Prevention, 2014a). We used the complete chromosome of CFSAN010068 as the seed genome to define a core genome MLST containing 2,685 genes. The resulting NJ tree showed a close relationship among food, clinical, and environmental isolates (Figure 2B), which helped indicate that the outbreak strain had spread across multiple zones and areas of the facility. The NJ tree also differentiated outbreak-associated isolates from a cheese isolate in New York (CFSAN009740) and a clinical isolate in California (PNUSAL000355), both of which were part of the initial epidemiological investigation (Chen et al., 2017a). The outbreak isolates differed by ≤ 13 alleles in pairwise comparisons with a maximum linkage of 5 alleles. cgMLST did not identify any polymorphic genes in either prophage (Table S3). Meanwhile, the CFSAN SNP Pipeline previously performed on this outbreak determined that the isolates differed by ≤ 12 SNPs with a maximum linkage of 5 SNPs (Chen et al., 2017a). The two prophages contained maximal 1 SNP (Table S3; Chen et al., 2017a).

#### 3. Multistate, Artisan Cheese, 2013 (Choi et al., 2014) (ST6, CC6, Serogroup IVb)

We used the complete genome of one outbreak isolate from cheese (CFSAN006122, NCBI Accession: CP007600.2; 2,802 protein-coding regions) and determined that the shotgun genomes of outbreak isolates contained 98.9–99.8% of the complete genome. The genes missing in those shotgun genomes were randomly scattered across the complete genome, indicating that shotgun-sequenced isolates did not lose any genomic islands that may be carried within CFSAN006122. This was consistent with our PHASTER analysis which predicted no prophages from CFSAN006122. No plasmid was identified in any isolate from either long-read- or shotgun-sequencing data.

**Figure 3:**
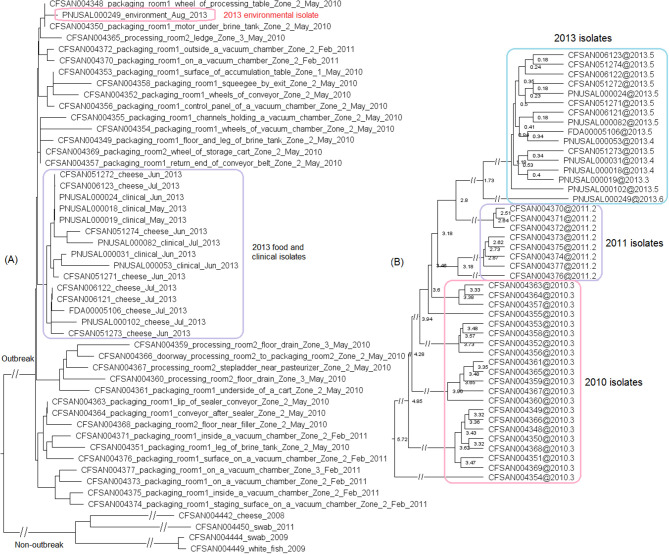
**(A)** NJ tree of selected available WGS data for the 2013 artisan cheese outbreak strain. All environmental isolates and a portion of representative food and clinical isolates are included in the tree. Environmental isolate ID is followed by facility location, zone information (when available), and isolation date. Clinical isolate ID is followed by the isolation date. Several isolates from cream cheese, fish and a fish processing facility serve as the non-outbreak outgroup, which shared the same PFGE profile as the outbreak isolates. The clade of 2013 food and clinical isolates is enclosed in the purple box and the 2013 environmental isolate is enclosed in the pink box. Isolates did not contain any prophage or plasmid. **(B)** Bayesian evolutionary analysis by sampling trees for the 2013 artisan cheese outbreak strain. The collection date of each isolate is following the symbol “@” which is following the isolate ID. The date is in decimal years (i.e., July 31, 2013 means 7 months or 0.6 year past the beginning of 2013, which is 2013.6). The number at each node is the time before the most recent isolation date (i.e., 2013.6). The 2013 isolates formed an exclusive monophyletic clade (enclosed in the blue box) inside the larger monophyletic clade that exclusively contained the 2011 (enclosed in the purple box) and 2013 isolates. The 2010 isolates are enclosed in the pink box.

The outbreak was recognized in 2013 and a facility was implicated. Subsequently, *Lm* isolated from the same facility in May 2010 and February 2011 during regular surveillance were subjected to shotgun sequencing. These environmental isolates were collected from various locations in Zones 1, 2, and 3, such as floors, drains, ladders, equipment wheels and legs, and processing equipment. We used the complete CFSAN006122 genome to define a core genome MLST containing 2,625 genes. In the resulting NJ tree, the 2013 isolates and the isolates collected in 2010 and 2011 were clustered together (Figure 3A), exhibiting ≤ 13 allelic differences with a maximum linkage of 7 alleles (Table S3). Meanwhile, the CFSAN SNP pipeline analysis determined that isolates differed by ≤ 10 SNPs with a maximum linkage of 3 SNPs (Table S3). This indicated that the outbreak strain had spread across multiple zones and areas of the facility.

Based on ESS values and nested sampling, the best model for BEAST analysis of these isolates was the strict clock model assuming a coalescent constant population tree prior. The average nucleotide substitution rate per year was 5.8 × 10^−7^ [95% highest posterior density (HPD) interval, 2.0 × 10^−7^ to 10.7 × 10^−7^] substitutions per site, or 1.7 substitutions per genome. In the BEAST tree (Figure 3B), the food and clinical isolates in 2013 and the only environmental isolate in 2013 formed a monophyletic clade, with the most recent common ancestor estimated to be present in November 2011 (95% HPD interval, October 2010 to January 2013). This clade and the 2011 isolates formed a larger monophyletic clade with the most recent common ancestor estimated to be present in June 2010 (95% HPD interval, March 2010 to September 2010). This provided a clue on the microevolution events of these isolates over the course of 3 years. For example, we could hypothesize that after positive *Lm* findings in 2010, sanitation practices might have eliminated most or all of the *Lm* in the facility; however, one or a few isolates might have survived or been reintroduced to the facility and diversified into the 2011 isolates. A similar hypothesis can be generated regarding the diversification of the 2013 isolates after the positive *Lm* findings in 2011. In contrast, the NJ tree placed the food and clinical isolates collected in 2013 into a monophyletic clade that is separate from 2013 environmental isolate (Figure 3A). In addition, the NJ tree did not place all 2011 isolates into one clade that is separated from 2010 isolates; thus, the NJ tree based on allelic profiles provided different clues on the microevolution of these isolates. This showed the value of performing in-depth SNP-based evolutionary analysis to identify microevolution events, which can contribute to root cause analysis.

#### 4. Multistate, Soft Cheese, 2010-2015 (Centers for Disease Control and Prevention, 2015b) (ST6, CC6, Serogroup IVb)

Complete genomes were available for two environmental isolates of this outbreak strain, CFSAN038814 (alternative ID, FDA00009448, chromosome NCBI Accession: CP044432.2; 2,942 protein-coding regions; plasmid NCBI Accession: CP044433.1, 65 genes) and FDA00006667 (chromosome NCBI Accession: CP044430.2; 2,920 protein-coding regions; plasmid NCBI Accession: CP044431.1, 65 genes). We determined that shotgun genomes of outbreak isolates contained 96.8–99.9% of the genes in the complete CFSAN038814 chromosome. Among them, shotgun genomes of 30 outbreak isolates contained 99.4–99.9% of the complete set of CFSAN038814 genes, and the genes missing in these genomes were randomly scattered across the chromosome; shotgun genomes of the other 8 outbreak isolates including FDA00006667 and the complete chromosome of FDA00006667 contained 96.8–97.7% of the complete set of CFSAN038814 genes. The difference was largely due to these genomes missing a 47 Kb region in CFSAN038814 (genome positions: 922427 to 969427, 72 genes, 2.4% of the complete gene set), which corresponded to part of CFSAN038814 prophage #1 predicted by PHASTER. This was consistent with our PHASTER and BLAST analysis. PHASTER predicted CFSAN038814 prophage #1 and CFSAN038814 prophage #2 (Tables S1, S2) in the complete CFSAN038814 genome, and predicted one prophage in the complete FDA00006667 genome (Table S2), which corresponded to a major part of CFSAN038814 prophage #2. BLAST analysis showed that 30 isolates contained CFSAN038814 prophage #1 and #2 and 8 isolates contained only CFSAN038814 prophage #2 (100% QC and >99.9% SI, Table S1). In contrast, PHASTER performed directly on shotgun genomes predicted two prophages in 29 isolates and one prophage in 9 isolates, and these predicted prophages all corresponded to large portions of CFSAN038814 prophage #1 or #2 (>99.9% SI), although the ends of each predicted prophage varied among different isolates, resulting in different prophage lengths (Table S1). BLAST alignment of CFSAN038814 prophages with shotgun genomes mostly confirmed PHASTER predictions from shotgun genomes except that CFSAN038814 prophage #1 could not be directly predicted from shotgun-sequenced PNUSAL001748 and the presence of prophage #1 in PNUSAL001748 was only determined by BLAST (Table S1).

The plasmid of CFSAN038814 was the same as the plasmid of FDA00006667 (100% QC and 100% SI) and was present in all 9 environmental isolates and 18 out of 29 clinical isolates (Table S1); nucleotide polymorphisms existed in 5 genes of the entire plasmid, including both IS6 family transposase. This plasmid contained *cadA2C2*, a gene cassette involved in *Lm* resistance to cadmium (Parsons et al., 2017).

**Figure 4:**
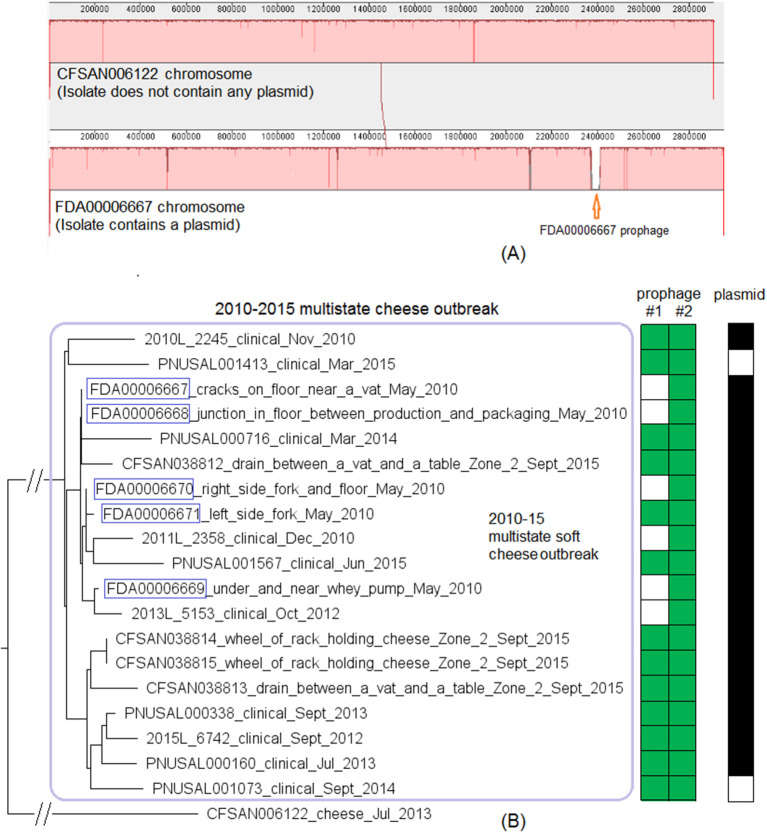
**(A)** Mauve alignment of the complete chromosome of CFSAN006122 from the 2013 artisan cheese outbreak and the complete chromosome of FDA00006667 from the 2010-2015 cheese outbreak, both of which were CC6. Same color in the blocks indicates homologous regions between the two strains, and the height of a specific region corresponds to similarity level of that region. The major difference in gene presence/absence was that CFSAN006122 did not contain the FDA00006667 prophage. The two strains also differed by presence/absence of a plasmid. **(B)** NJ tree of selected available WGS data for the 2010-2015 cheese outbreak strain (enclosed in the purple box). All environmental isolates and a portion of representative food and clinical isolates are included in the tree. Environmental isolate ID is followed by facility location, zone information (when available), and isolation date. Clinical isolate ID is followed by the isolation date. CFSAN006122 from the 2013 artisan cheese outbreak serves as the unrelated outgroup. The environmental isolates collected in 2010 (enclosed in blue boxes) fall into one clade. Two prophages (#1 and #2) were predicted and one plasmid was identified from these isolates. To the right of the tree, the green filled space indicates the presence of a prophage and the open space indicates the absence of a prophage. The black filled space indicates the presence of the plasmid and the open space indicates the absence of the plasmid. Prophage gain/loss did not correlate with phylogenetic clades.

We then compared FDA00006667 with the complete genome of a CC6 isolate (CFSAN006122) associated with the 2013 multistate soft cheese outbreak discussed in subsection 3 (Figure 4A). We found that CFSAN006122 contained 96.9% of the genes in FDA00006667, and most of the differences were due to CFSAN006122 missing the FDA00006667 prophage (containing 2.3% of the complete gene set). The 2013 multistate soft cheese outbreak strain did not contain any plasmid, suggesting plasmid gain/loss between the two outbreak strains.

This outbreak was recognized in 2015 and implicated a facility. Subsequently, epidemiological investigation identified case-patients diagnosed between 2010 and 2015 (Centers for Disease Control and Prevention, 2015b), and *Lm* isolates obtained through surveillance sampling in 2010 from the implicated facility were subjected to WGS. The environmental isolates collected in 2010 and 2015 were from facility locations such as floors, drains, floor cracks, forklifts, equipment wheels, and processing equipment. We used the CFSAN038814 complete chromosome to define a core genome MLST containing 2,739 genes. The resulting NJ tree clustered the clinical isolates with the environmental isolates collected in 2010 and 2015 (Figure 4B), and isolates differed by ≤ 30 alleles in pairwise comparisons with a maximum linkage of 14 alleles (Table S3) and with no polymorphic genes in the prophage. Isolates missing prophage #1 did not form any distinct clade. Meanwhile, the CFSAN SNP pipeline analysis showed that isolates differed by 32 SNPs with a maximum linkage of 16 SNPs. Prophages in CFSAN038814 contributed to maximal 5 SNPs (Table S3). The SNP tree was consistent with the NJ tree showing no exclusive clustering of isolates gaining/losing prophage #1 (Figure S2).

BEAST analysis conducted based on 29 isolates (Table S1) determined that the best model was the relaxed exponential clock assuming the coalescent Bayesian Skyline tree prior. The average substitution rate per year was 5.5 × 10^−7^ (95% HPD interval, 2.5 × 10^−7^ to 9.0 × 10^−7^) substitutions per nucleotide site, or 1.6 substitutions per genome. The most recent common ancestor of the outbreak strain was estimated to be in June 2006 (95% HPD interval, March 2001 to September 2009). The BEAST tree (not shown) generated a similar topology as the NJ and SNP trees regarding the major clusters. All 5 environmental isolates from the 2010 sampling fell into a clade within the outbreak cluster and were not in the ancestral positions of quite a few other isolates collected between 2010 and 2015 (Figure 4B, Figure S2). This indicated that these 5 isolates might represent only a portion of *Lm* population present in 2010.

#### 5. Multistate, Cantaloupe, 2011 (McCollum et al., 2013) (Strain #1, ST5, CC5, Serogroup IIb)

We used the complete genome of a clinical isolate (L2624, NCBI Accession: NZ_CP007686.1; 2,859 protein-coding genes) and determined that the shotgun genomes contained 99.1–99.7% of all genes in the complete L2624 genome. The genes missing in the shotgun genomes were randomly scattered across the L2624 genome, indicating that shotgun-sequenced isolates did not miss any genomic islands that may be carried within L2624. This is consistent with our PHASTER analysis which did not predict any prophages to be present in L2624 or any shotgun-sequenced isolates. No plasmid was identified in any isolate from either long-read sequencing or shotgun sequencing data.

**Figure 5:**
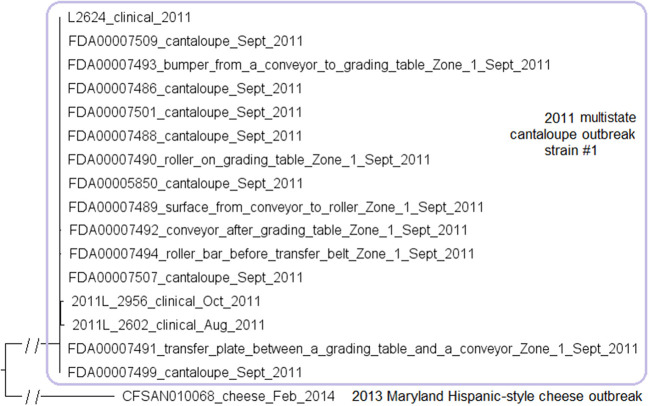
NJ tree of selected available WGS data for the 2011 multistate cantaloupe outbreak strain #1 (enclosed in the purple box). All environmental isolates and a portion of representative food and clinical isolates are included in the tree. Environmental isolate ID is followed by facility location, zone information (when available), and isolation date. Clinical and food isolate ID is followed by the source and isolation date. An unrelated CC5 isolate, CFSAN010068, from the 2013 Maryland cheese outbreak (subsection 2) serves as the outgroup. Isolates did not contain any prophage or plasmid.

The environmental isolates were collected in September 2011 from various Zone 1 locations, such as a conveyor and a roller, while the clinical isolates were collected between August and October 2011 (McCollum et al., 2013). We used the complete L2624 genome to define a core genome MLST containing 2,648 genes. The resulting NJ tree showed a close relationship among clinical and environmental isolates (Figure 5). The outbreak isolates differed by ≤ 9 alleles with a maximum linkage of 7 alleles (Table S3). Meanwhile, the CFSAN SNP Pipeline analysis determined that the outbreak isolates differed by ≤ 5 SNPs with a maximum linkage of 3 SNPs (Table S3).

#### 6. Multistate, Cantaloupe, 2011 (McCollum et al., 2013) (Strain #2, ST7 and Strain #3, ST561, CC7, Serogroup IIa)

The outbreak associated with contaminated cantaloupe in 2011 was a polyclonal outbreak. We analyzed the CC5 strain in the above subsection. Here, we analyzed strain #2 and #3 together because they both belonged to CC7, even though they had different STs. A complete genome was available for one isolate of each of the outbreak strains (i.e., outbreak strain #2, clinical isolate L2676, NCBI Accession: NZ_CP007685.1; 2,929 protein-coding regions, and outbreak strain #3, clinical isolate L2626, NCBI Accession: NZ_CP007684.1; 2,869 protein-coding regions). The shotgun genomes of different isolates of outbreak strain #2 contained 99.6–99.8% of the complete gene set of L2676, and the missing genes were randomly scattered across the L2676 genome. This was consistent with our PHASTER analysis that predicted two prophages in the complete L2676 genome and our BLAST analyses showing that all shotgun-sequenced isolates contained those two L2676 prophages (≥96% QC and ≥99.4% SI for prophage #1 and 100% QC and 100% SI for prophage #2) (Tables S1, S2). PHASTER analysis performed directly on shotgun genomes also predicted those two L2676 prophages and did not predict any additional prophage (Table S1).

The shotgun genomes of isolates of outbreak strain #3 contained 99.4–99.9% of genes in the complete L2626 genome. The missing genes were randomly scattered across L2626, which was consistent with our PHASTER analysis that predicted a prophage in the L2626 complete genome and our BLAST analysis showing that all shotgun-sequenced isolates contained that prophage (≥99% QC and 100% SI) (Tables S1, S2). PHASTER analysis performed directly on shotgun genomes also predicted the L2626 prophage and did not predict any other prophages (Table S1). No plasmid was identified in any isolate from either long-read sequencing or shotgun sequencing data.

We subsequently compared isolates of strain #2 and isolates of strain #3. The L2626 prophage was the same as the L2676 prophage #1 (Table S2) (100% SI with slightly different PHASTER-predicted ends). The major gene-scale differences were due to the isolates of strain #3 not having a 40 Kb island present in the isolates of strain #2 (L2676 genome position: 2361929–2402220, 57 genes between *comK* fragments), and this island corresponded to a major part of the L2676 prophage #2 (54 Kb). This prophage was located between two *Asc*I restriction sites (L2676 genome position: ~2063672 and ~2446932) and its absence resulted in the change of a 383 Kb fragment in outbreak strain #2 to a 343 Kb fragment in outbreak strain #3. This supports the previous hypothesis that prophage gain/loss caused the PFGE banding pattern changes between isolates of the two strains (Lomonaco et al., 2013).

**Figure 6:**
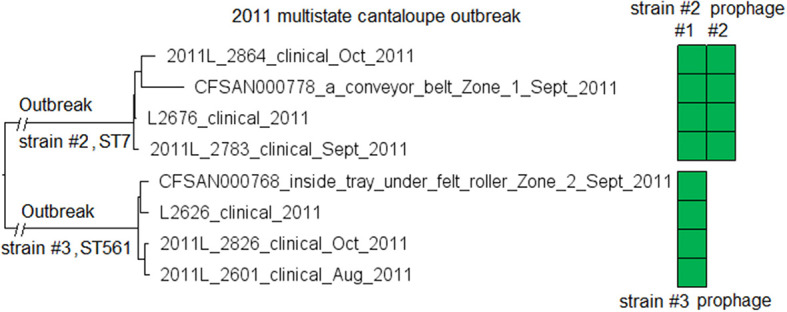
NJ tree of selected available WGS data for the 2011 multistate cantaloupe outbreak strain #2 and #3. All environmental isolates and a portion of representative clinical isolates are included in the tree. Environmental isolate ID is followed by facility location, zone information (when available), and isolation date. Clinical isolate ID is followed by the isolation date. Isolates of strain #2 contained two prophages (#1 and #2) and isolates of strain #3 contained one prophage. Prophage #1 of strain #2 and the prophage of strain #3 were the same (95% BLAST QC and 100% SI). Prophage #2 of strain #2 was not present in the isolates of strain #3. To the right of the tree, the green filled space indicates the presence of a prophage. Isolates did not contain any plasmid.

The environmental isolates were collected in September 2011 from several Zone 1 and Zone 2 locations, such as a conveyor belt and a tray under a roller, while the clinical isolates were collected between August and November 2011 (McCollum et al., 2013). We used the complete L2676 genome to define a core genome MLST containing 2,699 genes. The resulting NJ tree clearly separated outbreak strain #2 from strain #3 (141 to 153 allelic differences) (Figure 6). Within outbreak strain #2 and #3, isolates differed by up to 18 and 7 alleles, respectively, and the maximum linkage was 12 and 6 alleles, respectively (Table S3). Meanwhile, the CFSAN SNP Pipeline analysis showed that isolates of the outbreak strain #2 and #3 differed by 4 and 5 SNPs, respectively with a maximum linkage of 2 and 3 SNPs, respectively. There was no polymorphism in either prophages determined by cgMLST or SNP analysis (Table S3).

An environmental isolate collected from a conveyor belt inside the packaging area in Zone 1 (Biosample ID: CFSAN020644, Table S1) was ST11 of CC11. This isolate did not cluster with any clinical isolate. Therefore, in this facility, there were at least 4 different *Lm* strains from 3 CCs.

#### 7. Multistate, Caramel Apple, 2014-2015 (Angelo et al., 2017; Chen et al., 2017c) (Strain #1, ST1, CC1, Serogroup IVb) and Multistate, Soft Cheese, 2017 (Centers for Disease Control and Prevention, 2017) (ST1, CC1, Serogroup IVb)

**Figure 7:**
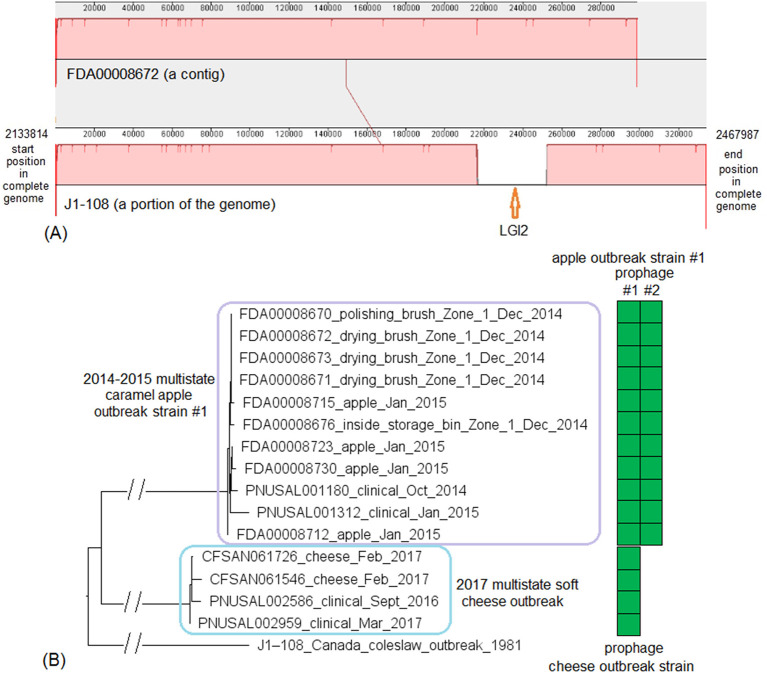
**(A)** Mauve alignment of corresponding chromosome regions between a contig of FDA00008672 (2014-2015 multistate caramel apple outbreak strain #1) and J1-108, both of which were CC1. The major differences of gene presence/absence were due to FDA00008672 not containing *Listeria* Genomic Island 2 (LGI2), which was present in J1-108. The two proteins upstream of this island in J1-108 were DNA repair exonuclease (locus tag, HK80_11665) and YlbF/YmcA family competence regulator (HK80_11670), and the two proteins downstream of this island were DUF445 family protein (HK80_11850) and class II fumarate hydratase (HK80_11855); these four genes were next to each other in the FDA00008672 genome. Neither isolates contained any plasmid. **(B)** NJ tree of selected available WGS data for the 2014-2015 caramel apple outbreak strain #1 (enclosed in the purple box) and the 2017 cheese outbreak strain (enclosed in the blue box). All environmental isolates and a portion of representative food and clinical isolates are included in the tree. Environmental isolate ID is followed by facility location, zone information (when available), and isolation date. Clinical isolate ID is followed by the isolation date. An unrelated CC1 isolate, J1-108, from a coleslaw outbreak in Canada serves as the outgroup. All isolates of the 2014-2015 caramel apple outbreak strain #1 contained two prophages (#1 and #2) and all isolates of the 2017 cheese outbreak strain contained one prophage, as indicated by the green and brown filled space to the right of the tree. Prophage #1 of the caramel apple outbreak strain #1 was the same as the prophage of the cheese outbreak strain. These prophages were different from the J1-108 prophage. Isolates did not contain any plasmid.

These two outbreak strains both belonged to CC1, and we analyzed them together. There were no complete genomes available for any isolates of these two strains, so we chose a complete genome from an unrelated outbreak strain, known to be CC1 (J1-108, NCBI Accession: CP006596.2; 2,892 protein-coding regions) for comparison with the caramel apple outbreak strain #1 and the 2017 cheese outbreak strain. The shotgun genomes from isolates of these two outbreak strains contained 95.1–96.1% of all genes in the complete J1-108 genome. Most of the genes missing in the shotgun genomes were in three regions of J1-108. First, the caramel apple outbreak strain #1 missed a 4 Kb putative islet (J1-108 genome position: 78574–82803, 7 genes); second, both outbreak strains lost a 39 Kb island (J1-108 genome position: 93703–132924, 61 genes), which was a major part of the prophage predicted to be present in J1-108 (Table S2); third, both outbreak strains lost a 36 Kb island (J1-108 genome position: 2349318–2385562, 37 genes) which we identified as the *Listeria* Genomic Island 2 (LGI2) (Figure 7A). Thus, the major gene-scale differences between J1-108 and these two outbreak strains were in the prophage, LGI2, or the putative islet. In fact, when we used the complete genome of another CC1 strain (F2365) without any prophage or LGI2 for comparison (Nelson et al., 2004), the caramel apple outbreak strain #1 and the cheese outbreak strain contained ~99.5% of genes in the complete F2365 genome.

PHASTER performed directly on shotgun genomes of the caramel apple outbreak strain #1 predicted prophage #1 (41 Kb) and prophage #2 (38–46 Kb, varying among different isolates) in all isolates (100% SI) (Tables S1, S4). PHASTER performed directly on shotgun genomes of the cheese outbreak strain predicted one prophage (41 Kb) in 9 of the 17 isolates (Table S4); and BLAST of this prophage against the other 8 isolates showed that those 8 isolates actually contained this prophage (≥95% QC and >99.9% SI), which was split into multiple contigs in the shotgun genomes. The two 41 Kb prophages from the two outbreak strains had 99.96% SI. Thus, the major gene-scale differences between the two outbreak strains were due to the gain/loss of prophage #2 of the caramel apple outbreak strain #1. No plasmid was identified from shotgun genomes.

For the caramel apple outbreak strain #1, environmental isolates were collected in December 2014 from Zone 1 locations, such as brushes and a storage bin, while clinical isolates were collected between October 2014 and January 2015 (Angelo et al., 2017). For the cheese outbreak strain, food isolates were collected in February 2017 and clinical isolates were collected between September 2016 and March 2017 (Centers for Disease Control and Prevention, 2017). We used the J1-108 complete genome to define a core genome MLST containing 2,699 genes. The resulting NJ tree showed a close relationship among the food and clinical isolates of the cheese outbreak strain and among the food, environmental, and clinical isolates of the caramel apple outbreak strain #1 (Figure 7B). Isolates of the caramel apple outbreak strain #1 differed by ≤ 8 alleles with a maximum linkage of 6 alleles; isolates of the cheese outbreak strain differed by ≤ 8 alleles with a maximum linkage of 6 alleles (Table S3). Meanwhile, the CFSAN SNP Pipeline analysis determined that isolates of the caramel apple outbreak strain #1 differed by ≤ 9 SNPs with a maximum linkage of 6 SNPs and that isolates of the cheese outbreak strain differed by ≤ 5 SNPs with a maximum linkage of 3 SNPs (Table S3).

#### 8. Multistate, Hispanic-Style Cheese, 2014 (Centers for Disease Control and Prevention, 2014b) (ST2 and ST1661, CC2, Serogroup IVb)

**Figure 8:**
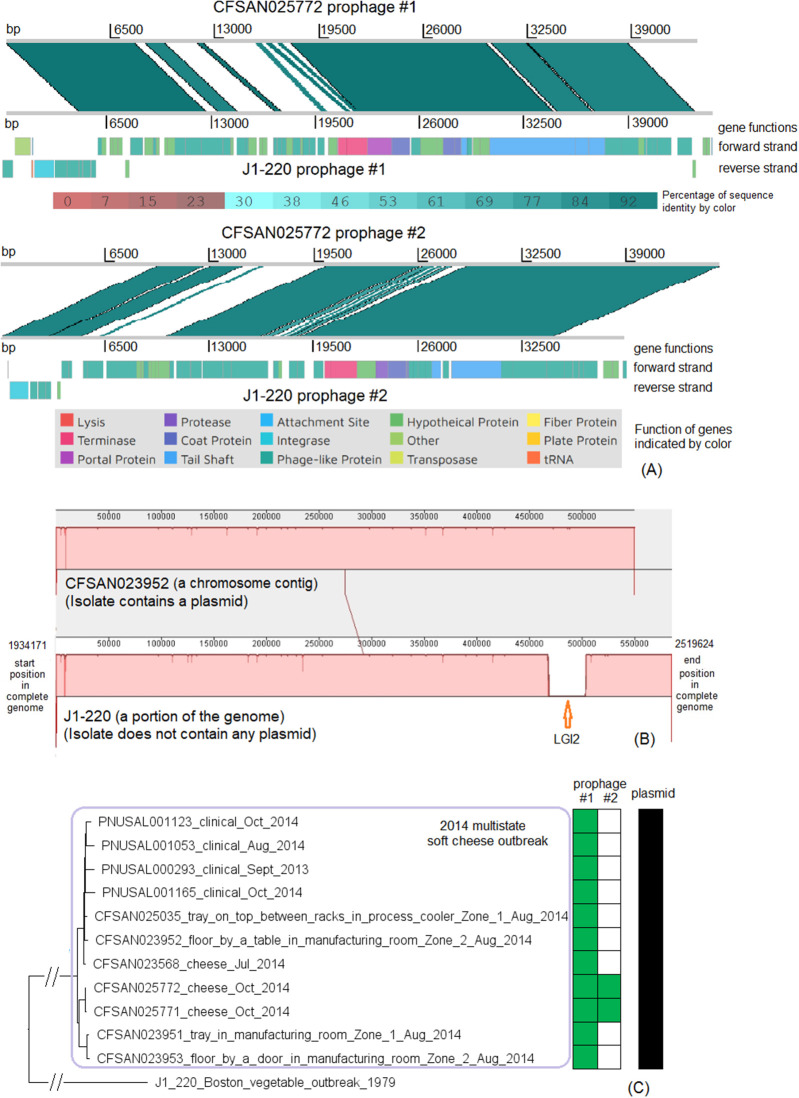
**(A)** Alignments between CFSAN025772 prophage #1 and J1-220 prophage #1, and between CFSAN025772 prophage #2 and J1-220 prophage #2, produced by Artemis Comparison Tool (ACT). The two strains both belonged to CC2, and J1-220 was from an unrelated vegetable outbreak in Boston. CFSAN025772 was chosen to represent the 2014 multistate cheese outbreak because it was one of the two isolates containing two prophages. The J1-220 prophages were predicted from the complete genome and the CFSAN025772 prophages were predicted from the shotgun genome. In the middle section of each alignment, the green color indicates DNA homology, determined by BLAST. Darker shades indicate higher sequence identities. Functions of major genes of J1-220 prophages were identified by PHASTER and color coded. In each comparison, two prophages shared homologous regions and diverse regions, suggesting the possibility of recombination events. **(B)** Mauve alignment of corresponding chromosome regions between a contig of CFSAN023952 and J1-220. CFSAN023952 was chosen to represent the 2014 multistate soft cheese outbreak because its contig corresponding to the J1-220 LGI2 region was relatively long. The major difference in gene presence/absence was due to CFSAN023952 not containing LGI2. The two proteins upstream of LGI2 in J1-220 were DNA repair exonuclease (locus tag, 10317) and YlbF/YmcA family competence regulator (locus tag, 10322), and the two proteins downstream of this island were DUF445 family protein (locus tag, 19520) and class II fumarate hydratase (locus tag, 10332); these four genes were next to each other in the CFSAN023952 contig. CFSAN023952 contained a plasmid and J1-220 did not contain any plasmid. **(C)** NJ tree of selected available WGS data for the 2014 multistate cheese outbreak strain (enclosed in the purple box). All environmental isolates and a portion of representative clinical isolates were included in the tree. Environmental isolate ID is followed by facility location, zone information (when available), and isolation date. Clinical isolate ID is followed by the isolation date. J1-220 serves as the outgroup. Two prophages (#1 and #2) were predicted from isolates of the 2014 cheese outbreak strain. To the right of the tree, the green filled space indicates the presence of a prophage, and the open space indicates the absence of a prophage. Isolates containing prophage #2 formed a clade; they were ST1661 in contrast to ST2 of other isolates of the outbreak. All isolates contained a plasmid, as illustrated by the black filled space to the right of the tree.

There were no complete genomes available for this 2014 outbreak strain. We selected an archival strain of the same CC for which a complete genome was on file (J1-220, NCBI Accession: CP006046.4; 2,951 protein-coding regions) for comparison. Two prophages (#1 and #2) were predicted from J1-220 (Table S2). PHASTER performed directly on shotgun genomes predicted prophage #1 (43–44 Kb) and #2 (45 Kb) (Table S4). Prophage #1 was present in all isolates (100% QC and 100% SI), and prophage #2 was present in 2 of 11 isolates (100% QC and 100% SI) (Table S1). Multiple recombination events may have contributed to the differences between prophage #1 of the cheese outbreak strain and J1-220 prophage #1 (Table S2) (77% QC and 98% SI with BLAST matches in separate ranges); hypervariable regions were mostly in genes encoding hypothetical proteins, phage proteins, and terminases (Figure 8A). A similar observation was made between prophage #2 of the cheese outbreak strain and J1-220 prophage #2 (Table S2) (75% QC and 91% SI with matches in separate ranges); hypervariable regions were mostly in genes encoding hypothetical proteins and phage proteins (Figure 8A). Nonetheless, we cannot exclude the possibility of a single prophage replacement event that led to these variations. The 2014 cheese outbreak strain lost an entire region in J1-220 (position in the genome: 2401169–2437413, 37 genes) which we identified as LGI2 (Figure 8B). Other than these regions, the shotgun genomes of isolates of the 2014 cheese outbreak strain only missed ~0.5% of the complete gene set of J1-220. Together, these findings demonstrate that the major difference among isolates of the cheese outbreak strain was the gain/loss of prophage #2, and the major gene-scale differences between the cheese outbreak strain and J1-220 were due to recombination or replacement of prophage(s) and gain/loss of LGI2. All isolates of the 2014 cheese outbreak strain possessed a plasmid, which contained *cadA1C1*. In contrast, no plasmid was found in J1-220 from either long-read sequencing or shotgun sequencing data, indicating plasmid gain/loss between the two strains.

The environmental isolates were collected in August 2014 from locations in Zone 1 and Zone 2, such as floors and trays, the cheese isolates were collected in July and October 2014, and the clinical isolates were collected between September 2013 and October 2014 (Centers for Disease Control and Prevention, 2014b). We used the complete J1-220 genome to define a core genome MLST containing 2,732 genes. In the resulting NJ tree, all outbreak isolates were clustered together (Figure 8B), with ≤ 21 allelic differences and a maximum linkage of 11 alleles (Table S3). Isolates that contained prophage #2 were ST1661, different from other isolates (ST2), and formed a clade inside the cluster of all outbreak isolates (Figure 8C). Meanwhile, the CFSAN SNP Pipeline analysis determined that the outbreak-associated isolates differed by ≤ 21 SNPs with a maximum linkage of 13 SNPs (Table S3). SNP-based phylogenetic analysis was consistent with the cgMLST analysis in placing isolates that contained prophage #2 into one clade inside the outbreak cluster (Figure S3).

#### 9. Multistate, Caramel Apple (Strain #2), 2014-2015 (Angelo et al., 2017; Chen et al., 2017c) Multistate, Stone Fruit, 2014 (Jackson et al., 2015; Chen et al., 2016a); Multistate, Leafy Green, 2015-2016 (Centers for Disease Control Prevention, 2016; Chen et al., 2017c) (Singleton ST382, Serogroup IVb-v1)

Here we combined multiple outbreak strains, all belonging to singleton ST382, into one analysis because we only had one complete genome available out of these outbreak strains. We also included a set of ST382 isolates from cheese, environmental, and clinical samples collected in an incident initially suspected as an outbreak, though further investigation determined that cheese was not the vehicle of the incident. We analyzed this set of isolates to see if our analysis generated results consistent with epidemiological investigations.

We used the complete genome of one isolate from the stone fruit outbreak (CFSAN023463, NCBI Accession: CP012021.2; 2,829 protein-coding regions) and determined that shotgun genomes contained 99.0–99.8% of the genes in the complete CFSAN023463 genome with only 3 exceptions. These exceptions were 3 shotgun genomes that contained slightly less (98.2–98.6%) of the genes in the CFSAN023463 genome. The genes missing in the shotgun genomes were randomly scattered across the complete CFSAN023463 genome, indicating that the shotgun genomes did not miss any genomic islands that may be carried within the CFSAN023463 genome. This was consistent with our PHASTER analysis showing that no prophage was predicted to be present in CFSAN023463. In addition, no plasmid was identified in any isolate from either long-read sequencing or shotgun sequencing data.

**Figure 9:**
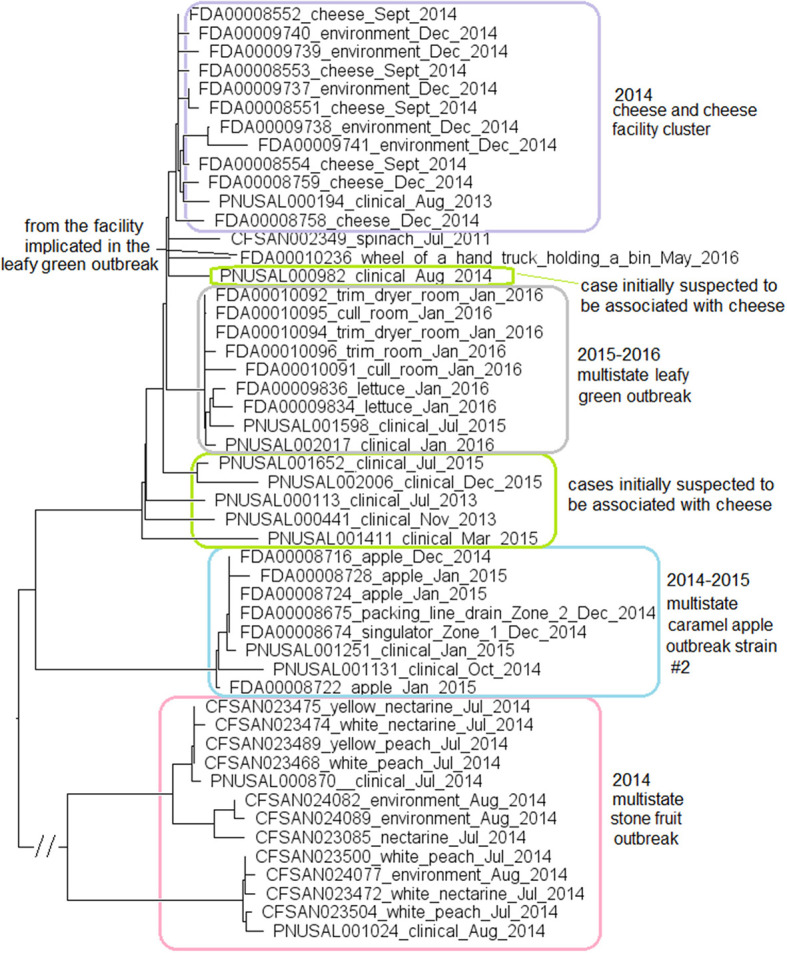
NJ tree of selected available WGS data for the ST382 isolates, the 2015-2016 leafy green outbreak (enclosed in the gray box), the 2014-2015 caramel apple outbreak strain #2 (enclosed in the blue box), the 2014 stone fruit outbreak (enclosed in the pink box), the 2014 cheese and facility cluster (enclosed in the purple box) and the cases initially suspected to be associated with the cheese (enclosed in green boxes). All environmental isolates and a portion of representative food and clinical isolates are included in the tree. Environmental isolate ID is followed by facility location, zone information (when available), and isolation date. Clinical isolate ID is followed by the isolation date. Food isolate is followed by the food type and isolation date. Isolates did not contain any prophage or plasmid.

For the stone fruit outbreak strain, the food and environmental isolates were collected in July and August 2014 (Jackson et al., 2015). For the caramel apple outbreak strain #2, environmental isolates were collected in December 2014 from different Zone 1 and Zone 2 locations, such as a drain and processing equipment, while the clinical isolates were collected between October and December 2014 (McCollum et al., 2013). For the leafy green outbreak strain, the environmental isolates were collected in January 2016 from multiple locations, while the food and clinical isolates were collected between July 2015 and January 2016 (Centers for Disease Control and Prevention, 2016). We used the CFSAN023463 complete genome to define a core genome MLST containing 2,632 genes. In the resulting NJ tree, the stone fruit, caramel apple, and leafy green outbreak isolates formed three monophyletic clusters that corresponded to each outbreak (Figure 9), which also indicated that the outbreak strain had spread across multiple areas in each facility. The isolates of caramel apple outbreak strain #2, the stone fruit outbreak strain, and the leafy green salad outbreak strain differed by up to 11, 38, and 11 alleles, respectively, and their maximum linkage was 6, 30, and 5 alleles, respectively (Table S3); previously performed CFSAN SNP Pipeline analysis showed that isolates of these outbreak strains differed by up to 4, 42, and 7 SNPs, respectively, and their maximum linkage was 2, 35, and 5 SNPs, respectively (Table S3) (Chen et al., 2016a, 2017c).

In May 2016, an isolate (Biosample ID: FDA00010236) was isolated from the facility implicated in the leafy green outbreak and differed from the leafy green outbreak isolates by 10 to 17 alleles; however, this isolate did not belong to the outbreak cluster (Figure 9). Two other strains were collected from the facility implicated in the stone fruit outbreak, CC5 (discussed below in subsection 11) and singleton ST392 (Biosample ID: CFSAN024093), indicating multi-clone contamination of the facility.

Among isolates collected during the investigation of a suspected outbreak, isolates from cheese products, the cheese production facility and one patient (Biosample ID: PNUSAL000194) formed a monophyletic cluster. Epidemiological investigation did not show that the patient consumed the implicated brand of cheese. Other clinical isolates were outside the cheese cluster (Figure 9). Thus, our results were consistent with the epidemiological investigation concluding that the cheese was not the vehicle for the illnesses.

#### 10. Multistate, Ice Cream, 2010-2015 (Chen et al., 2017b) (Strain#1 in Facility #1, ST5, CC5, Serogroup IIb; and Strain #2 in Facility #2, ST5, CC5, Serogroup IIb, Same Firm). Florida, Ice Cream, 2017 (Allard et al., 2019) (ST5, CC5, Serogroup IIb)

Here we combined three CC5 outbreak strains from two outbreaks associated with ice cream into one analysis because we only had one complete genome available out of these outbreak strains (CFSAN029793, CP016213.2; 2,957 protein-coding regions). The Florida outbreak involved patients in an assisted living facility (Allard et al., 2019). The multistate ice cream outbreak involved two outbreak strains, strain #1 and strain #2, associated with two ice cream production facilities, facility #1 and #2, respectively (Chen et al., 2017b). CFSAN029793 was an isolate of strain #1. In addition, we also included two non-outbreak CC5 isolates (PNUSAL001431 and PNUSAL001433) collected from ice cream produced in facility #1; the ice cream yielding outbreak strain #1 and those yielding the non-outbreak isolates were from different production lines of this facility. The shotgun-sequenced isolates of these outbreak strains contained 95.0–99.0% of the complete gene set of CFSAN029793. Other than the regions described below, the shotgun genomes only missed ~0.5% of the genes in the complete genome, and the missing genes were randomly scattered across the genome.

Comparing the shotgun-sequenced isolates of multistate outbreak strain #1 with the complete CFSAN029793 genome, most of the genes missing were in 4 regions and each region was absent in a portion of the shotgun genomes (Table S1). First, a 4 Kb putative islet (CFSAN029793 genome position: 76494–80557, 6 genes); second, a 43 Kb region (position in CFSAN029793 genome: 1257652–1300951, 60 genes) which corresponded to a major part of prophage #1 predicted by PHASTER to be present in the complete CFSAN029793 genome (Table S2); third, a 40 Kb region (position in CFSAN029793 genome: 2408872–2449198, 58 genes), which corresponded to a major part of CFSAN029793 prophage #2 (Table S2); and fourth, a 38 Kb region (position in CFSAN029793 genome: 2587819–2626053, 56 genes), which corresponded to a major part of CFSAN029793 prophage #3 (Table S2).

**Figure 10:**
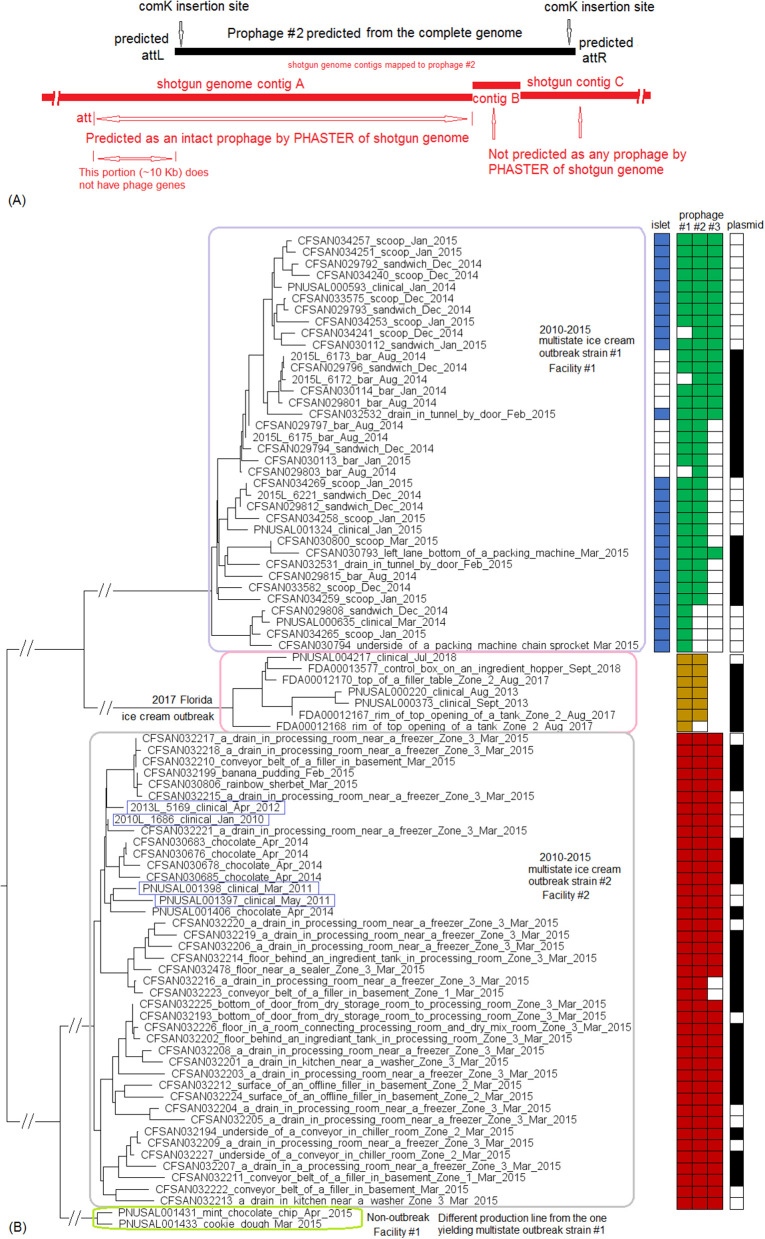
**(A)** Comparison between prophage #2 predicted from the CFSAN029793 complete genome and the PHASTER prediction from the CFSAN029793 shotgun genome. The figure is for illustration purposes and the lengths of genomic contigs or regions are not necessarily proportional to their actual lengths. CFSAN029793 prophage #2 was a *comK* prophage and the PHASTER prediction was consistent with insertion in *comK*. This prophage was split into 3 contigs in the shotgun genome with contigs B and C not predicted as any prophage, and part of contig A predicted as an intact prophage. This intact prophage included a ~10 Kb region that did not contain any prophage genes and was upstream of the CFSAN029793 prophage #2; this ~10 Kb region was included in this intact prophage possibly because the phage attachment site was predicted to be at the beginning of the ~10 Kb region. **(B)** NJ tree of selected available WGS data for the 2015 multistate ice cream outbreak strain #1 (enclosed in the purple box), outbreak strain #2 (enclosed in the gray box), and the 2017 Florida ice cream outbreak strain (enclosed in the pink box). All environmental isolates and a portion of representative food and clinical isolates are included in the tree. Environmental isolate ID is followed by facility location, zone information (when available), and isolation date. Clinical isolate ID is followed by the isolation date. Food isolate is followed by the food type and food production date. Three prophages were predicted from multistate ice cream outbreak strain #1, three prophages were predicted from multistate outbreak strain #2 and two prophages were predicted from the 2017 Florida ice cream outbreak strain. A 4 Kb islet was also missing in a portion of isolates of multistate ice cream outbreak strain #1. To the right side of the tree, the blue filled space indicates the presence of the islet, the green/brown/red filled space indicates the presence of a prophage, the black filled space indicates the presence of the plasmid, and the open space indicates the absence of a prophage, islet, or plasmid. Prophage #1 of one outbreak strain was different from prophage #1 in another outbreak strain, so were prophage #2, prophage #3, and the plasmids among different outbreak strains. Prophage #2 of multistate outbreak strain #1 was the same as prophage #3 of multistate outbreak strain #2. Prophage gain/loss partially correlated with phylogenetic clades. For example, for multistate ice cream outbreak strain #1, isolates missing prophage #2 formed a clade. Isolates of strain #1 missing the plasmid formed three clades. The isolates of the multistate ice cream outbreak strain #2 were divided into two major clades and the four isolates collected prior to 2014 (enclosed in blue boxes) fall into one clade within the outbreak cluster.

PHASTER analysis performed directly on the shotgun genomes could not predict some CFSAN029793 prophages in certain isolates, even when BLAST analysis showed that those prophages were actually present in the isolates (≥95% QC and >99.7% SI for all prophages). In some other isolates, prophages predicted by PHASTER from shotgun genomes corresponded to large portions (i.e., 70 to 95%) of CFSAN029793 prophages, but PHASTER could not predict the entire prophages (Tables S1, S2). An example of the inadequacy of prophage prediction directly from shotgun genomes is illustrated in Figure 10A.

PHASTER analysis performed directly on the shotgun genomes of isolates of the multistate ice cream outbreak strain #2 predicted three prophages with the ends of each prophage varying slightly among different isolates (Tables S1, S4). Combined PHASTER and BLAST analyses showed that prophage #1 was present in all isolates (≥99% QC and >99.9% SI), prophage #2 was present in all isolates (≥96% QC and >99.8% SI), and prophage #3 was present in all but two isolates (≥95% QC and >99.7% SI). Thus, the major gene-scale differences among isolates of the multistate outbreak strain #2 were due to the gain/loss of prophage #3.

PHASTER analysis performed directly on the shotgun genomes of isolates of the Florida ice cream outbreak strain predicted two prophages with the prophage ends varying slightly among different isolates (Tables S1, S4). Combined PHASTER and BLAST analyses showed that prophage #1 was present in all isolates (≥97% QC and >99.9% SI), and prophage #2 was present in all but one isolate (≥98% QC and >99.9% SI). Thus, the major gene-scale differences among isolates of this outbreak strain were due to the gain/loss of prophage #2.

Comparing the multistate outbreak strain #1 and multistate outbreak strain #2, prophage #1 of each strain was unique to that strain; prophage #2 of strain #1 was the same as prophage #3 of strain #2 (93% QC and 99.99% SI); and recombination or prophage replacement may have contributed to the differences between prophage #3 of strain #1 and prophage #2 of strain #2 (55% QC and 92% SI with matches in separate ranges). Comparing the multistate outbreak strain #1 and the Florida outbreak strain, recombination or prophage replacement may have contributed to the differences between prophage #1 of strain #1 and prophage #2 of the Florida strain (62% QC and 92% SI with BLAST matches in separate ranges) and the differences between prophage #3 of strain #1 and prophage #1 of the Florida strain (47% QC and 92% SI with matches in separate ranges). This indicated that the major gene-scale differences among these three strains were in the prophages.

Comparison between published complete plasmid sequences and shotgun genomes, including the shotgun genome of CFSAN029793, revealed that 22 out of 42 isolates (52.4%) of the multistate outbreak strain #1 analyzed in this study contained a plasmid, which contained *cadA2C2*; no plasmid was identified in any of the 4 clinical isolates or 16 out of 38 of food and environmental isolates. Similarly, 27 out of 43 isolates (62.8%) of the multistate outbreak strain #2 contained a plasmid, which possessed *cadA1C1*; no plasmid was identified in the 5 clinical isolates or 11 out of 38 food and environmental isolates. Among the isolates of the Florida outbreak strain, 6 out of 7 isolates (85.7%) possessed a plasmid, which contained *bcrABC*, a gene cassette involved in benzalkonium chloride (BC) tolerance, and *cadA1C1*; one clinical isolate did not possess the plasmid.

For the multistate ice cream outbreak strain #1, environmental isolates were collected in February and March 2015 from separate facility locations, such as drains and bottoms of processing equipment; food isolates were collected from samples produced between December 2014 and March 2015; and clinical isolates were collected between January 2014 and January 2015 (Centers for Disease Control and Prevention, 2015a). For the multistate ice cream outbreak strain #2, environmental isolates were collected in March 2015 from various locations in Zones 1, 2, and 3, such as drains, floors, conveyor belts, and processing equipment; food isolates were collected from samples produced between April 2014 and March 2015; and clinical isolates were collected between January 2010 and November 2014 (Centers for Disease Control and Prevention, 2015a). For the Florida outbreak strain, environmental isolates were collected between August 2017 and September 2018 from various Zone 2 locations outside of processing equipment, while clinical isolates were collected between August 2013 and July 2018 (Allard et al., 2019). We used the CFSAN029793 complete genome to define a core genome MLST containing 2,717 genes. The resulting NJ tree clustered isolates belonging to individual outbreak strains (Figure 10B), indicating that outbreak strains had spread to multiple locations of implemented facilities.

Isolates of the multistate ice cream outbreak strain #1 differed by ≤ 30 alleles with a maximum linkage of 14 alleles. cgMLST identified one polymorphic gene in the three CFSAN029793 prophages (Table S3). Previously performed CFSAN SNP Pipeline analysis determined that the isolates differed by ≤ 29 SNPs with a maximum linkage of 16 SNPs. CFSAN029793 prophages contributed to maximal 2 SNPs (Table S3) (Chen et al., 2017b). Many isolates having the same patterns regarding the presence/absence of prophage/islet fell into the same clades; however, there were many exceptions (Figure 10B). Therefore, the gain/loss of prophages only partially correlated with the nucleotide variations in the genome backbone, which was also observed in previously performed SNP-based phylogenetic analysis (Burall et al., 2017; Chen et al., 2017b). However, SNP-based phylogeny identified a 13-isolate clade with good bootstrap support, containing 11 of the 12 isolates from ice cream bars, and was suggested to be strongly associated with ice cream bars (Chen et al., 2017b); this is consistent with the cgMLST analysis of only isolates of multistate outbreak strain #1 (Figure S4). In contrast, such clade was not observed in the cgMLST analysis combining all three outbreak strains together (Figure 10B).

Isolates of the multistate outbreak strain #2 differed by ≤ 26 alleles, with a maximum linkage of 15 alleles (Table S3). Previously performed CFSAN SNP Pipeline analysis determined that the isolates differed by ≤ 29 SNPs with a maximum linkage of 13 SNPs (Table S3) (Chen et al., 2017b). The two isolates that lost prophage #3 formed one clade (Figure 10B), consistent with the SNP-based analysis (Chen et al., 2017b).

The non-outbreak CC5 strain, collected in a facility #1 production line different from the one in which the ice cream yielding outbreak strain #1 was produced, differed from outbreak strain #1 by 244 to 254 alleles, and differed from outbreak strain #2 by 34 to 53 alleles, a result consistent with the SNP-based analysis (Chen et al., 2017b). This indicated that the two different production lines in facility #1 were contaminated with two different CC5 strains. These findings demonstrated how WGS can differentiate among isolates obtained from different facilities, isolates obtained from different production lines of the same facility or even isolates from different product types produced in the same production line.

Isolates associated with the Florida outbreak differed by ≤ 28 alleles with a maximum lineage of 15 alleles (Table S3). Meanwhile, the CFSAN SNP Pipeline analysis determined that the isolates differed by ≤ 20 SNPs with a maximum linkage of 15 SNPs (Table S3). Although another cluster of CC224 isolates was identified from multiple Zone 1 and Zone 2 locations (e.g., processing equipment, side of a container and pump) of the implicated ice cream production facility, those isolates were not associated with any clinical cases (Allard et al., 2019).

BEAST analysis was conducted on the 2010-2015 multistate outbreak strain #2 because the isolates (Table S1) were collected over 5 years. The two models, relaxed lognormal clock and strict clock, when the coalescent Bayesian Skyline population tree prior was assumed, yielded nearly identical clock rate and nearly identical marginal likelihoods in nested sampling which were statistically higher than the marginal likelihoods generated using other model and tree prior combinations. The average mutation rate per year was 4.5 × 10^−7^ (95% HPD interval, 2.5 × 10^−7^ to 6.6 × 10^−7^) substitutions per nucleotide site, or 1.4 substitutions per genome. The most recent common ancestor of the outbreak strain was estimated to be in March 2007 (95% HPD interval, September 2003 to January 2010). Even though there are some differences in the topology among the BEAST tree (tree not shown), the NJ tree, and the SNP tree (Chen et al., 2017b), they all suggested two major clades with the four isolates collected between 2010 and 2012 falling into one clade (Figure 10B); these isolates were not in the ancestral position of quite a few other isolates collected between 2014 and 2015. This indicated that these four isolates might represent only a portion of *Lm* population present in 2010.

#### 11. CC5 Isolates From Stone Fruit and Production Environment of the Facility Linked to the 2014 Stone Fruit Outbreak but Not Associated With the Illnesses

We analyzed CC5 isolates from stone fruits and their production facility collected during the investigation of the 2014 stone fruit outbreak (Chen et al., 2016a). Even though these isolates were not linked to any clinical cases, they represented another cluster of isolates from the implicated food and food processing environment. We used the complete genome of one of these CC5 isolates for analysis (CFSAN023459, chromosome NCBI Accession: CP014252.2; 2,993 protein-coding regions; one plasmid pCFSAN023459_01, NCBI Accession: NZ_CP014253.1, 17 genes, and the other plasmid pCFSAN023459_02, NCBI Accession: NZ_CP014254.1, 63 genes). The shotgun genomes of isolates from the stone fruit and their production facility contained 99.4–99.8% of the genes in the complete CFSAN023459 chromosome. The genes missing in the shotgun genomes were randomly scattered across the CFSAN023459 chromosome. This was consistent with our PHASTER and BLAST analyses, which showed that all stone fruit and facility isolates contained the three prophages predicted to be present in CFSAN023459 (≥96% QC and >99.9% SI, 100% QC and 100% SI, and ≥94% QC and >99.4% SI, for prophage #1, #2, and #3, respectively) (Tables S1, S2). PHASTER performed directly on shotgun genomes revealed another prophage (herein designated as stone fruit CC5 prophage #4), present in 16 out of 20 isolates (≥98% QC and >99.9% SI). This prophage was not present in CFSAN023459, as determined using its complete and shotgun genomes. Therefore, the major chromosomal gene-scale differences among these isolates were due to the gain/loss of stone fruit CC5 prophage #4. The plasmid pCFSAN023459_01 was present in all isolates except CFSAN024084, and the plasmid pCFSAN023459_02 was present in all isolates. Nucleotide variations existed in two genes of pCFSAN023459_01 and in two genes of pCFSAN023459_02.

**Figure 11:**
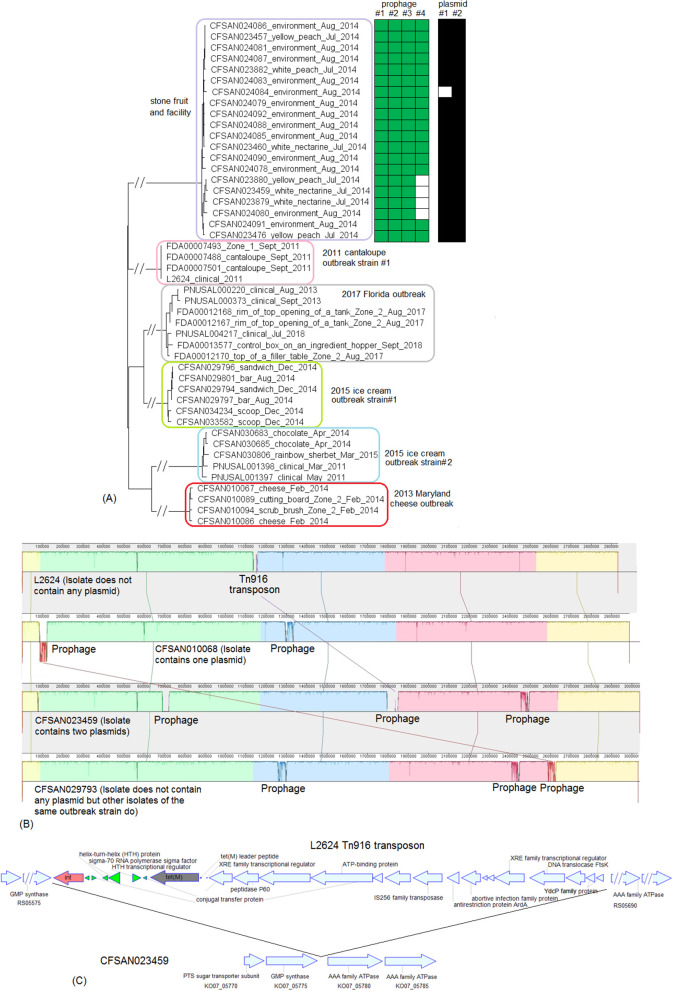
**(A)** NJ tree of selected available WGS data for CC5 isolates involved in outbreaks and recalls. All environmental isolates and a portion of representative food and clinical isolates are included in the tree. Environmental isolate ID is followed by facility location, zone information (when available), and isolation date. Clinical isolate ID is followed by the isolation date. Food isolate is followed by the food type and isolation date. From these isolates, four prophages (#1, #2, #3, and #4) were predicted and one two plasmids were identified. To the right of the tree, the green filled space indicates the presence of a prophage, black filled space indicated the presence of a plasmid, and the open space indicates the absence of a prophage or plasmid. **(B)** Mauve alignment of the four complete CC5 chromosomes (L2624, CFSAN010068, CFSAN023459, and CFSAN029793) showing that major differences in presence/absence of genes were due to the gain/loss of the Tn916 transposon and prophages. CFSAN010068 contained a plasmid, which was substantially different from the two CFSAN023459 plasmids (BLAST QC ≤ 15%). **(C)** Genomic organization of the Tn916-like transposon in L2624. Gene functions annotated by NCBI and protein locus tags of flanking proteins are shown. The upstream proteins were PTS sugar transporter subunit and GMP synthase. The two downstream proteins were AAA family ATPase. CFSAN023459 did not have Tn916 inserted in its corresponding region. Inside the transposon, the red arrow indicates the integrase, the gray arrow indicates the tetracycline resistance gene, light blue arrows indicate the conjugal related proteins, and green arrows indicate insertion genes and regulation. Hypothetical proteins or proteins with unknown functions are not labeled.

We used the CFSAN023459 chromosome to define a core genome MLST containing 2,734 genes. Environmental isolates from multiple locations of the facility were clustered with stone fruit isolates (Figure 11A); isolates differed by ≤ 17 allelic differences with a maximum linkage of 9 alleles. Prophages contributed to maximal 3 allelic differences (Table S3). The 4 isolates missing prophage #4 formed a monophyletic clade. Previously performed CFSAN SNP Pipeline analysis showed that isolates differed by ≤ 17 SNPs with a maximum linkage of 9 SNPs (Table S3) (Chen et al., 2016a). There was one polymorphic site in all prophages and the 4 isolates missing prophage #4 also formed a monophyletic clade (Chen et al., 2016a). We also brought together multiple sets of CC5 strains which have all been associated with listeriosis outbreaks, and isolates belonging to each outbreak strain were all correctly clustered (Figure 11A). Interestingly, the two produce-associated strains formed a large clade and the four dairy-associated strains formed another large clade (Figure 11A). More CC5 isolates could be analyzed to determine whether WGS clusters could be attributed to food sources.

We then performed a comparison among all the complete chromosomes of CC5: CFSAN029793, representing the 2010-2015 multistate ice cream outbreak strain #1; L2624, representing the 2011 multistate cantaloupe outbreak strain #1; CFSAN010068, representing the 2013 Maryland cheese outbreak strain; and CFSAN023459, representing the non-outbreak strain isolated during investigation of the 2014 multistate stone fruit outbreak. Mauve alignment (Figure 11B) showed that the major differences were the gain/loss of two CFSAN010068 prophages, three CFSAN029793 prophages, three CFSAN023459 prophages, and a 22 Kb genomic island in L2624 (L2624 genome position: 1136523 to 1158094, 25 genes) which we identified as a Tn916-like transposon (Figure 11C). Therefore, most chromosomal gene-scale differences among these 4 strains were in the prophage and transposon regions.

We subsequently compared the CC5 outbreak strains with CFSAN010068 plasmid, pCFSAN010068_01 (55 genes, 56 Kb, discussed in subsection 2) and CFSAN023459 plasmids, pCFSAN023459_01 (17 genes, 13 Kb) and pCFSAN023459_02 (63 genes, 53 Kb). pCFSAN023459_01 and the plasmids of the 2010-2015 multistate ice cream strain #1, strain #2 or the 2017 Florida ice cream outbreak strain were totally different (i.e., BLAST QC <10% when pCFSAN023459_01 was aligned to the shotgun genome). pCFSAN010068_01 contained 7 of 17 genes of pCFSAN023459_01, suggesting possible recombination events occurring to the two plasmids, but pCFSAN010068_01 was substantially longer. The plasmid-carrying isolates of the 2013 Maryland cheese outbreak strain and 2010-2015 multistate ice cream outbreak strain #1 contained 13 and 33 genes of pCFSAN023459_02 and BLAST of pCFSAN023459_02 against these two outbreak strains had 20 and 32% QC, suggesting possible recombination events occurring to the plasmids of the two outbreak strains and pCFSAN023459_02. pCFSAN023459_02 and the plasmids of 2010-2015 multistate ice cream outbreak strain #2, and the 2017 Florida ice cream outbreak strain were totally different (i.e., <8% BLAST QC when pCFSAN023459_02 was aligned to the shotgun genomes). The plasmid-carrying isolates of the 2010-2015 multistate ice cream outbreak strain #1, #2, and the Florida outbreak strain contained 17, 25, and 37 of 55 genes of pCFSAN010068_01, respectively, and BLAST of the pCFSAN010068_01 against these strains had QC of 34, 22, and 24%, indicating possible recombination events occurring to the plasmids of these outbreak strains and pCFSAN010068_01. Therefore, these CC5 strains differed by gain/loss/recombination of plasmids.

### Overall Analysis

#### Long-Read Sequencing Offered Advantages in Identifying MGEs, Which Accounted for Most of the Gene-Scale Differences Among Isolates of the Same Outbreak Strain, and Among Different Outbreak Strains of the Same CC

In this study, we analyzed isolates of multiple outbreak strains using available long-read and shotgun WGS data. Data from long-read sequencing identified MGEs such as prophages, *Listeria* genomic island 2, Tn916-like transposon, and plasmids. BLAST was used to determine whether these were present in shotgun-sequenced isolates. We attempted to predict prophages directly from shotgun genomes using PHASTER and found that such prediction was not as accurate as that performed on complete genomes. When a complete prophage was split into multiple contigs of a shotgun genome, PHASTER could locate a large portion of the prophage in some isolates and could not predict any prophage in other isolates (Table S1). On the other hand, prophage ends predicted by PHASTER from complete genomes were not always consistent with prophage insertion sites, evidenced by the prediction of *comK* prophages in this study (e.g., L2676 prophage #2 that was predicted to be 54 Kb by PHASTER but only 40 Kb was inside the *comK* insertion sites). Nonetheless, when long-read sequencing data are not available, prophage prediction from shotgun genomes could provide useful information. In our study, we used CLC Genomics Workbench 11 assembly method with its default settings. Further improvement on shotgun sequencing and assembly methods could increase the accuracy of prophage prediction from shotgun genomes. BLAST using existing plasmids allowed identification of plasmid contigs in shotgun genomes, but long-read sequencing was needed to close the entire plasmid. When PacBio sequencing did not reveal a plasmid in an isolate, we also could not identify a plasmid in its shotgun genome, confirming the results of PacBio sequencing.

Our data showed that during short-term evolutions (i.e., among isolates of the same outbreak strain), *Lm* diversified by accumulating limited single nucleotide polymorphisms in the entire genome and by gaining/losing chromosome-borne prophages and plasmids. We did not investigate possible plasmid-borne prophages. Prophage regions constituted 2–5% of the complete chromosome. Other than prophages, genes missing in the shotgun genomes, when compared to the complete chromosome of the same outbreak strain, constituted ~0.5% of the genes in the complete chromosome and were randomly scattered across the chromosome. The incomplete prophages designated by PHASTER did not contribute to significant gene-scale differences among isolates of the same outbreak strain, confirming that questionable and intact prophages predicted by PHASTER should be used for prophage analysis. During medium- to long-term evolutions, such as those involving different strains of the same CC, the major gene-scale differences were in prophages, a Tn916-like transposon, and LGI2 among isolates analyzed in the present study. Recombination in prophages or prophage replacements frequently occurred to different strains of the same CC, resulting in a large amount of nucleotide variations. Similarly, a previous study demonstrated that during long-term evolutions, such as those involving multiple CCs, serotypes, and genetic lineages, the major gene-scale differences were in hypervariable hotspots and MGEs such as prophages, transposons, and mobile genomic islands (Kuenne et al., 2013). When different lineages of *Lm* were compared, mobile genetic elements represented one of the major categories of *Lm* accessory genome (den Bakker et al., 2013).

The LGI2 in J1-220 (CC2) and J1-108 (CC1) were nearly identical (100% BLAST QC and 99.93% SI); they both contained 36 genes, including an integrase, a sigma factor, an ABC transporter, conjugal transfer genes, a LPXTG cell wall anchor, and several genes involved in resistance to arsenic and cadmium (Lee et al., 2017). The flanking regions of LGI2 in J1-220 and J1-108 were also nearly identical, with DNA repair exonuclease and YlbF/YmcA family competence regulator at the upstream and DUF445 family protein and class II fumarate hydratase at the downstream. This island was initially identified by Kuenne et al. (2013) and was later found to be in a few CC1, CC2, and CC4 strains (Lee et al., 2017). The flanking regions of the Tn916-like transposon in L2624 were PTS sugar transporter subunit IIBC and GMP synthase at the upstream and ATP-dependent helicase and AAA family ATPase at the downstream. It contained integrase, tetracycline resistance ribosomal protection protein (TetM), and conjugal transfer proteins (Figure 11C) (Dong et al., 2014).

Interestingly, for certain outbreak strains, plasmid loss occurred more frequently in clinical isolates than food and environmental isolates. Specifically, for the 2013 Maryland cheese outbreak strain, 1 of 5 clinical isolates lost the plasmid, while 1 of 48 food and environmental lost the plasmid. For the 2010-2015 multistate cheese outbreak strain, 11 of 29 clinical isolates lost the plasmid, and all 9 environmental isolates contained the plasmid. For the 2010-2015 multistate ice cream outbreak strain #1, all 4 clinical isolates lost the plasmid and 16 of 38 food and environmental isolates lost the plasmid. For the 2010-2015 multistate ice cream outbreak strain #2, all 5 clinical isolates lost the plasmid and 11 of 38 food and environmental isolates lost the plasmid. For the 2017 Florida ice cream outbreak strain, 1 of 3 clinical isolates lost the plasmid and no environmental isolates lost the plasmid. The outbreak strains in this study were mostly lineage I isolates. In another study, plasmids were found more frequently in lineage II food isolates than lineage II clinical isolates (Pirone-Davies et al., 2018). Plasmids encode important proteins that can help *Lm* tolerate various stress (Hingston et al., 2019), and this could explain the presence of plasmids in food and environmental isolates. In contrast, our comparison was performed between clinical and food/environmental isolates of the same outbreak strain. It is possible that isolates without plasmids had alternative mechanisms of stress tolerance, although it is also possible that isolates lost plasmid(s) during transmission from food to human. The mechanisms underlying our results could warrant further investigations.

In addition to the MGEs described above, 2 to 6 genes in each of the two IS3-like transposons (Kuenne et al., 2013) in CFSAN010068 (7 genes: CG42_02655 to CG42_02685, 6 genes: CG42_10795 to CG42_10820) were missing in all shotgun genomes of the 2013 Maryland cheese outbreak strain. This occurred in other outbreak strains as well, including 2 to 4 genes in two IS3-like transposons in CFSAN006122 (7 genes: Y193_13415 to Y193_13385, and 6 genes: Y93_05435 to Y93_05410), 2 to 6 genes in each of two IS3-like transposons in L2624 (8 genes: RS02445 to RS02480, and 7 genes: RS10410 to RS10440), 3 to 4 genes in each of two IS3-like transposons (8 genes: VV80_14985 to VV80_15020, and 7 genes: VV80_07940 to VV80_07970) of CFSAN029793. We believe these were artifacts of shotgun sequencing or assembly due to repeat sequences, because (1) among the strains described in our study, when certain shotgun-sequenced isolates of an outbreak strain missed a MGE, we never observed that all shotgun-sequenced isolates missed that MGE, and (2) whenever an outbreak strain had IS3-like transposons, we observed several genes in the transposon missing in shotgun genomes; thus, we speculated that the chance of this phenomenon genuinely happening was very low.

#### Strain-Specific cgMLST Improved Resolution of WGS Analyses

In this study, we did not attempt to use a pre-defined cut-off value to define outbreak isolates. Instead, we chose isolates that were epidemiologically linked to an outbreak and employed WGS to show that the food, environmental, and clinical isolates of the same outbreak formed a monophyletic cluster (Pightling et al., 2018). We then determined the WGS diversity of these isolates. We used strain-specific cgMLST and whole genome SNP analysis and both methods consistently clustered isolates of the same outbreak strain. The genetic distances among isolates associated with each outbreak determined by the two methods were generally similar (Table S3). In contrast to species-specific cgMLST schemes that typically target 1,700 to 1,900 genes (Ruppitsch et al., 2015; Chen et al., 2016b; Moura et al., 2016; Jagadeesan et al., 2019), this scheme would target 2,600 to 2,800 genes, thus maximizing the discriminatory power. In addition, this scheme could be used to simultaneously analyze multiple outbreak strains of one CC because a cgMLST scheme could provide accurate clustering of isolates if those isolates contain >95% or even >90% of the cgMLST target set (Ruppitsch et al., 2015) and different strains of one CC analyzed in this study all shared >95% genes. A whole genome MLST scheme targeting 4,797 loci chosen from a pangenome of 150 previously published genomes (Chen et al., 2017a; Jagadeesan et al., 2019) would provide similar discriminatory power because ~2,700 to~2,900 loci may be targeted when a specific outbreak strain is analyzed. However, strain-specific cgMLST could include any functional loci that are unique to any novel strains. The advantage of *Lm* cgMLST over strain-specific cgMLST is that cgMLST targeting the entire *Lm* population allows standardized comparison of genetic diversity of isolates from different outbreak strains. When we analyzed all the outbreak strains in this study using the previously developed 1827-gene cgMLST targeting the entire population of *Lm* (Chen et al., 2016b), we found that the maximum pairwise allelic differences among isolates of the same outbreak strain was 20 alleles and the maximum linkage was 9 alleles except in the stone fruit outbreak (Table S3). As also suggested in other studies, WGS diversity was valuable as a starting point for an outbreak investigation, but WGS data should be interpreted in the context of any additional supporting and epidemiological data of a specific outbreak (Kwong et al., 2016; Chen et al., 2017a; Nielsen et al., 2017; Jagadeesan et al., 2019).

CFSAN SNP Pipeline targets the whole genome, including intergenic regions (Davis et al., 2015), which are not analyzed in cgMLST-based analysis. In addition, it was argued that reference-based mapping could be more accurate than *de novo* assembly-based methods, especially in repeat regions (Kwong et al., 2016). In addition, allelic profile is a combination of numbers and thus only distance-based methods can be used for phylogenetic analyses. In contrast, SNP-based methods can integrate robust statistical algorithms and evolutionary models. For example, bootstrap analysis (Efron et al., 1996) can be used to assess the confidence in individual phylogenetic clades; such analysis was not possible with allelic profiles.

A key feature in any WGS analysis is to overcome any bias introduced by recombination or sequencing artifacts to phylogeny reconstruction. Allele-based methods count multiple nucleotide variations in the same gene as one allele change to overcome such bias. Similarly, CFSAN SNP Pipeline employs a filter to remove nucleotide variations that may be due to recombination or sequencing artifacts. The default setting was to exclude an entire region which contained ≥3 SNPs in any 1,000 bp span between any two isolates from the final SNP matrix, even when other isolates in the same analysis contained <3 SNPs in this region. A limitation of such filtering occurs when distantly related and closely related strains are included in one single analysis, because distantly related isolates could contain ≥3 true SNPs in any 1,000 bp region; excluding such region from the final SNP matrix would also underestimate diversity of e.g., closely related isolates that may contain <3 true SNPs in this region. Indeed, when multiple CC5 strains from different outbreaks were included in a single CFSAN SNP Pipeline analysis, the WGS diversity determined for isolates associated with an outbreak was less than the diversity determined when the CFSAN SNP Pipeline was performed exclusively on isolates associated with that outbreak (Chen et al., 2017b). Thus, in our study, after we performed the SNP analysis to show separation between outbreak isolates and unrelated isolates, we performed a second SNP analysis to precisely determine SNP distance among outbreak isolates.

A complete WGS pipeline often involves multiple steps, such as raw reads trimming, *de novo* assembly, reference mapping, BLAST, SNP filtering, and phylogeny reconstruction. Multiple software and parameters are available for each step of the analysis, making direct comparison of different pipelines difficult (Lüth et al., 2018). Other whole genome sequencing tools were also developed, such as k-mer based hqSNP Pipelines (Jagadeesan et al., 2019) and JSpecies tetranucleotide analysis (Burall et al., 2016). A further evaluation of different tools using the outbreak strains in this study could facilitate a global standardization of WGS analysis (Lüth et al., 2018).

#### Gain/Loss of Prophages Did Not Compromise the Accurate Clustering of Outbreak-Associated Isolates If Properly Developed WGS Tools Were Used

Prophage regions and other MGEs, including plasmids, were highly conserved among isolates of the same outbreak strain, as evidenced by the low diversity. Initially we set BLAST SI to >98% to determine the presence of a prophage in an isolate, but our data subsequently showed that the actual BLAST SI was all above 99.4%. In our cgMLST analysis, we used the “pairwise ignore missing values” setting; thus, if two isolates both contained a prophage, any variations in the prophage were counted during allelic difference calculations. CFSAN SNP Pipeline also included SNPs in a prophage if this prophage was present in a portion of the isolates. When the reference genome and shotgun genomes were from the same outbreak strain, true SNPs in the prophages were not filtered out due to low genetic diversity, except that some false positive high-density SNPs in repetitive regions near the prophages ends were filtered out (as discussed below). Because of this limited diversity, prophages did not lead to inaccurate phylogenetic clustering among related and unrelated isolates. A study on *Salmonella* showed that the chromosomal MGEs had limited impact on the SNP diversity among isolates of the same outbreak strain, while plasmids could generate high-density SNPs (Li et al., 2019). Analyses of additional outbreaks could offer more insights on the effect of MGE on WGS analysis during traceback investigations.

**Figure 12:**
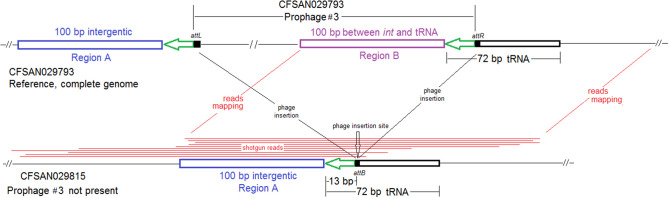
Illustration of shotgun reads of CFSAN029815 mapped to the complete CFSAN029793 genome near CFSAN029793 prophage #3. The figure is for illustration purposes and the lengths of genomic contigs or regions are not necessarily proportional to their actual lengths. CFSAN029815 did not have this prophage. In CFSAN029815, a 100 bp intergenic region A (blue open box) was upstream of the 72 bp tRNA. The phage insertion disrupted the tRNA at the *attB* core cross section site (black filled box). The phage contained a sequence identical to the 3' end of the tRNA (green open arrow, 13 bp) and a 100 bp intergenic region B (purple open box) between this 13 bp sequence (green open arrow) and *int*. As a result, after phage insertion the entire tRNA remained the same between CFSAN029793 and CFSAN29815. Regions A and B differed by 20 SNPs, so region A plus the 3' end of the tRNA and region B plus the 3' end of the tRNA formed a repeat in CFSAN029793. In an ideal scenario, when raw reads (red lines) of CFSAN029815 were mapped to CFSAN029793, raw reads of CFSAN029815 region A should map to region A of CFSAN029793; raw reads of CFSAN029815 tRNA should map to tRNA of CFSAN029793; and no reads from CFSAN029815 should map to any regions inside CFSAN029793 prophage #3. However, genomic DNA were randomly fragmented during shotgun sequencing, and for MiSeq sequencing V2, the reads can be 200 to 300 bp long. Some 200–300 bp raw reads of CFSAN029815 spanned region A and the tRNA. When these reads were mapped to the CFSAN029793 genome, they were mapped to region B and tRNA of CFSAN029793 because these differed by only 20 SNPs and were otherwise identical. Subsequent SNP analysis would call those 20 SNPs. These were false SNPs because CFSAN029815 did not contain region B.

Although not investigated in this study, repetitive DNA regions are known to cause artifacts to *de novo* assembly-based methods (Lüth et al., 2018). In previous studies using the CFSAN SNP Pipeline, repetitive regions led to high-density SNPs (Chen et al., 2016a, 2017a,b,c). Prophage ends are known to contain repeats, and Figure 12 provides an example of the 2010-2015 multistate ice cream outbreak strain #1 where reference mapping of repetitive sequences near the ends of a prophage generated false, high-density SNPs when prophage gain/loss occurred between the reference genome and the shotgun genome. These false SNPs were filtered out by the SNP Pipeline. If such SNPs were in coding regions, cgMLST would have counted them as one allelic difference per gene. Therefore, both methods could overcome the bias introduced by the artifacts caused by repeat sequences. When different outbreak strains were compared, recombination events that may have occurred to prophages could result in thousands of nucleotide variations in one prophage, and these SNPs could get filtered out when CFSAN SNP Pipeline was performed. In contrast, cgMLST would count these variations, and thus, cgMLST performed on a group of distantly related isolates may offer increased resolution over CFSAN SNP Pipeline.

Isolates belonging to different outbreak strains typically exhibited distinct prophage profiles (Figure 11B). Interestingly, we observed that for some outbreak strains, isolates gaining or losing a prophage fell into one clade, indicating that prophage gain/loss correlated with SNPs in genome backbones (Figures 6, 8C, 10B, 11A).

#### Utility of WGS on the Analysis of Polyclonal Outbreaks

The multistate outbreaks associated with cantaloupe, caramel apple, and ice cream described above were typical examples of polyclonal outbreaks resulting from multi-strain or multi-clone contamination in foods and food processing environments. The outbreak associated with cantaloupes had 5 outbreak strains (McCollum et al., 2013; Chen et al., 2016b), 3 of which were isolated from cantaloupes or their processing environments and were discussed in this study. Among them, strain #1 belonged to a serotype different from strain #2 or #3. Stain #2 and #3 belonged to the same CC and differed by 141 to 153 alleles in 2,641 genes (i.e., 2,699 genes of strain #2 minus 58 prophage genes missing in strain #3). The two outbreak strains associated with caramel apples belonged to different serotypes. The two CC5 outbreak strains associated with the ice cream differed by 203 to 238 alleles; this difference was consistent with previous studies showing that the two strains differed by ≤ 123 alleles using a 1748-gene cgMLST (Gerner-Smidt et al., 2019) and by 241 to 272 whole genome SNPs (Chen et al., 2017b). These examples illustrate how WGS can accurately match clinical isolates with food and environmental isolates for each individual outbreak strain in a polyclonal outbreak. Within the scope of one outbreak, the number of different outbreak strains and the genetic distance among different outbreak strains demonstrate the complexity of the contamination event(s), but do not compromise the utility of WGS during traceback investigations.

#### The Same *Lm* Strain Can Be Repeatedly Isolated From Multiple Locations and Zones of Food Processing Environments and WGS Data Can Help Generate Hypotheses on Microevolution Events

Our analyses demonstrated that outbreak strains could be distributed in multiple locations and zones of food production environments. Further, these strains can be persistent or repeatedly reintroduced in a facility over the course of several months to multiple years. The locations where *Lm* were frequently recovered were floors, drains, pallets, and equipment legs and wheels.

WGS analyses could also provide valuable clues that help generate hypotheses about the microevolution of the environmental isolates in a facility, which could contribute to root-cause analysis. In the 2010-2015 multistate ice cream outbreak, WGS differentiated isolates from two facilities, and within facility #1, WGS distinguished isolates from two production lines. Interestingly, the NJ tree (Figure 10) based on a single cgMLST analysis of all 3 outbreak strains did not identify the clade strongly associated with ice cream bars; this clade was identified by the cgMLST analysis of only isolates of outbreak strain #1 (Figure S4) and by the SNP analysis with strong bootstrap support (91%). This illustrated the importance of subjecting isolates of individual outbreak strains to separate analyses so that microevolution events can be discovered inside each outbreak cluster. In general, we believe the SNP analysis was more accurate due to the utilization of evolutionary models rather than distance-based clustering (e.g., NJ clustering). Similarly, Bayesian evolutionary analysis based on SNPs among isolates collected over 3 years in the facility implicated in the 2013 artisan cheese outbreak suggested how isolates collected from each year might have diversified into isolates collected in the following years. Therefore, to study microevolution of isolates, it is valuable to perform both *de novo* assembly- and SNP-based analyses and focus on isolates of the same WGS cluster.

In our study, we performed BEAST analysis on three outbreaks in which isolates had been isolated over 3 years because such heterochronous data enabled relatively confidant estimation of evolutionary rates and dates of the most recent common ancestor. Two outbreaks were associated with cheese products contaminated by CC6. Isolates in each outbreak had different genetic diversity and their evolution were best explained by different molecular clock models and tree priors, but the average substitutions per nucleotide site per year were similar, 5.8 × 10^−7^ and 5.5 × 10^−7^ for the 2013 artisan cheese outbreak and the 2010-2015 soft cheese outbreak, respectively. The isolates of the 2010-2015 multistate ice cream strain #2 had 4.5 × 10^−7^ substitutions per nucleotide site per year. These rates were higher than the average rates (i.e., 2.5 × 10^−7^ per site per year) of CC1 and CC9 collected from different sources over more than 90 years. Outbreaks represented short-term evolution scenarios and it is possible that isolates involved in outbreaks evolved faster than the average population.

#### Presence of Selected Genes Associated With Virulence and Stress Resistance

**Figure 13:**
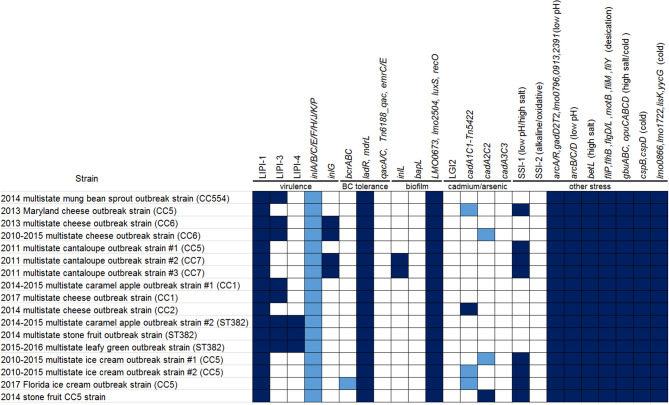
Presence (filled space) and absence (open space) of selected virulence and stress resistance genes of strains analyzed in this study. Dark blue filled spaces indicate that all isolates of the strain contained the genes. Light blue filled spaces indicate that not all isolates of the strain contained the genes. One category of such genes included chromosome-borne internalin genes (detailed results of each individual internalin gene not shown). The other category of such genes included plasmid-borne *bcrABC* and cadmium resistance cassettes because of the plasmid missing among a portion of isolates of each outbreak strain.

The presence of major virulence genes and genes implicated in stress response and environmental persistence (Moura et al., 2016; Maury et al., 2019) were determined. *Listeria* Pathogenicity Island 1 (LIPI-1) was present in all the isolates. LIPI-3 was present in CC554, CC6, CC1, and ST382. LIPI-4, was found only in the three ST382 outbreak strains and the non-outbreak ST382 strain from cheese. ST382 was associated with three produce-associated multistate outbreaks; therefore, these ST382 strains could be hypervirulent. Isolates contained major internalins (*inlABCEFHJKP*) with few exceptions. The stress survival islet 1 (SSI-1), involved in tolerance of low pH and high salt concentrations, was present in only CC5 and CC7 isolates, not any serotype 4b strains. SSI-2, involved in tolerance of alkaline and oxidative stress, was not found in any isolates (Figure 13). All the plasmid-carrying isolates contained one of the two known plasmid-borne cadmium resistance cassettes, *cadA1C1*, or *cadA2C2*. These isolates included 4 of the 5 CC5 outbreak strains, the non-outbreak CC5 strain from stone fruit and their packing facility, 1 of the 2 CC6 outbreak strains and the CC2 outbreak strain. Among all the plasmid-carrying isolates, only isolates of the Florida ice cream outbreak strain carried *bcrABC*, a BC resistance cassette. Due to the gain/loss of plasmid(s), the plasmid-borne genes were not always present in all isolates of an outbreak strain (Figure 13). Identification of markers for virulence and persistence could contribute to future functional analysis, however simple presence/absence of genes may need to be combined with gene expression data to best interpret phenotypes (Nielsen et al., 2017).

## Conclusions

In this study, we used a combination of complete genomes and strain-specific cgMLST to analyze MGEs among *Lm* isolates associated with select listeriosis outbreaks and to study the microevolution of *Lm* isolates in food processing environments. Our demonstration of leveraging archival sequences from multiple foodborne outbreaks illustrates the greater resolution of WGS analyses targeting the entire genome and shows that major gene-scale differences during both short-term and long-term evolution of *Lm* were in MGEs.

## Data Availability Statement

All datasets analyzed for this study are included in the article/Supplementary Materials.

## Author Contributions

HY wrote the manuscript and analyzed the data. MH helped with the data analysis. MA and EB provided scientific advisement. YC wrote the manuscript, analyzed the data, and conceptualized the study.

## Conflict of Interest

The authors declare that the research was conducted in the absence of any commercial or financial relationships that could be construed as a potential conflict of interest.
