# Supplementary figures and images for: Microevolution and Gain or Loss of Mobile Genetic Elements of Outbreak-Related Listeria monocytogenes in Food Processing Environments Identified by Whole Genome Sequencing Analysis
Source: Front Microbiol. 2020 May 29;11:866. doi: 10.3389/fmicb.2020.00866 (PMC7272582; doi:10.3389/fmicb.2020.00866)

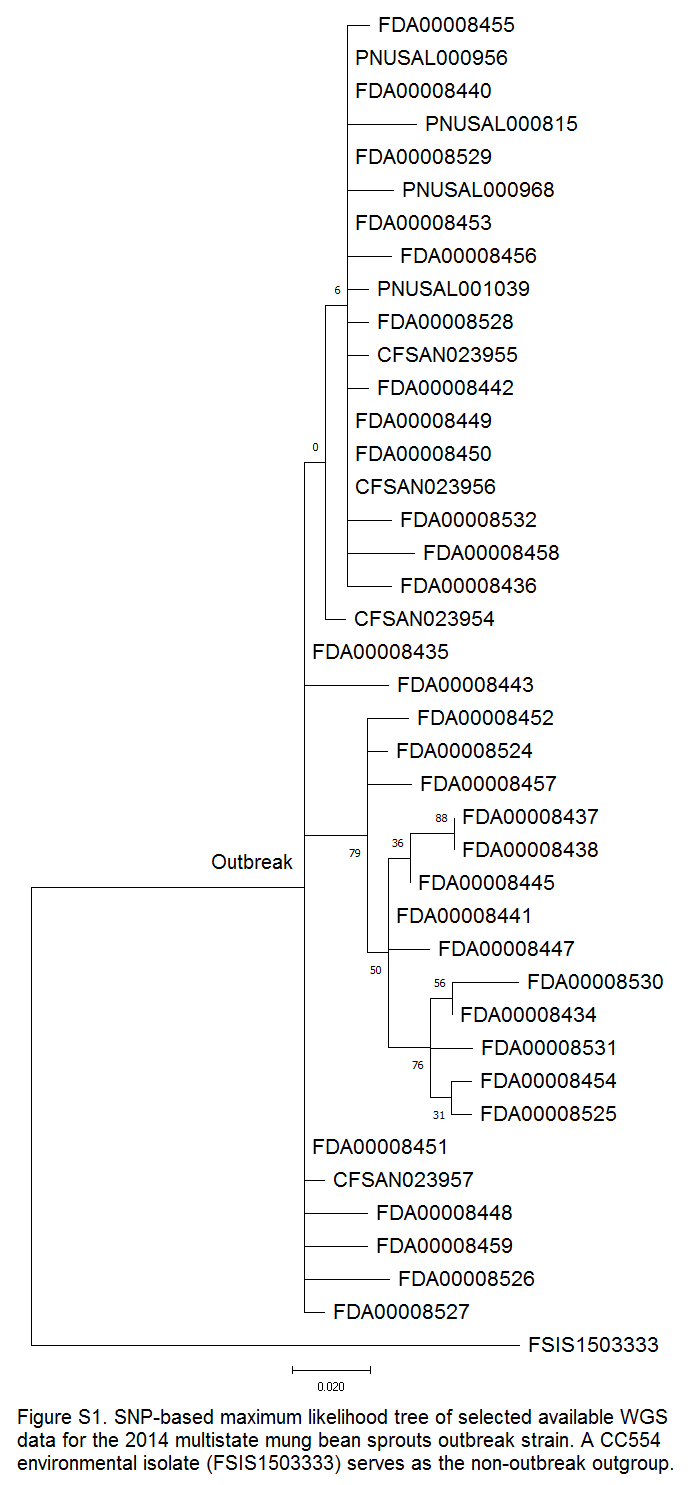

Supplement: Supplementary file 5 [file Image_1.TIF]

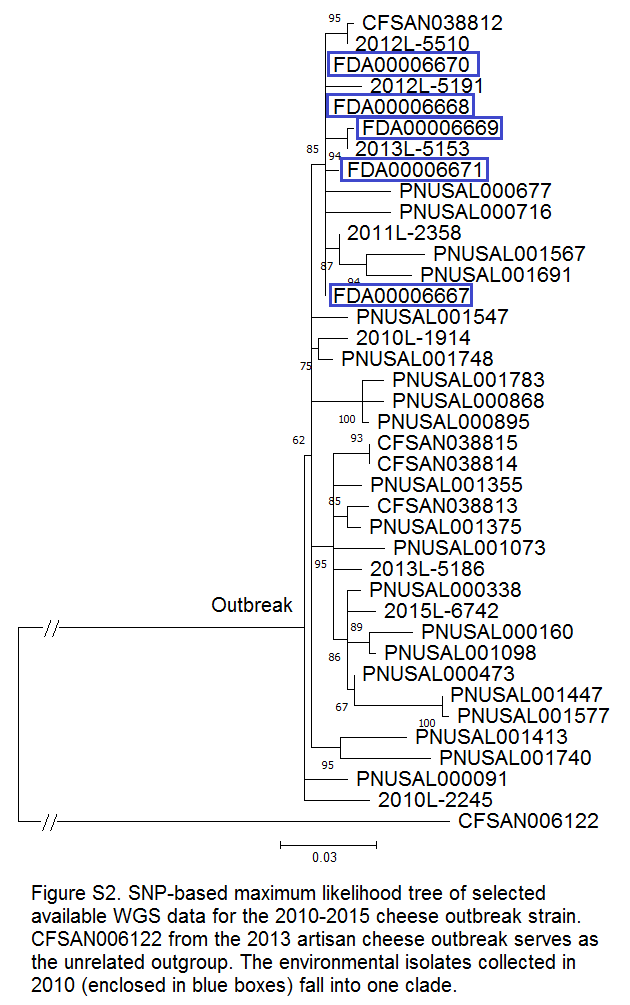

Supplement: Supplementary file 6 [file Image_2.TIF]

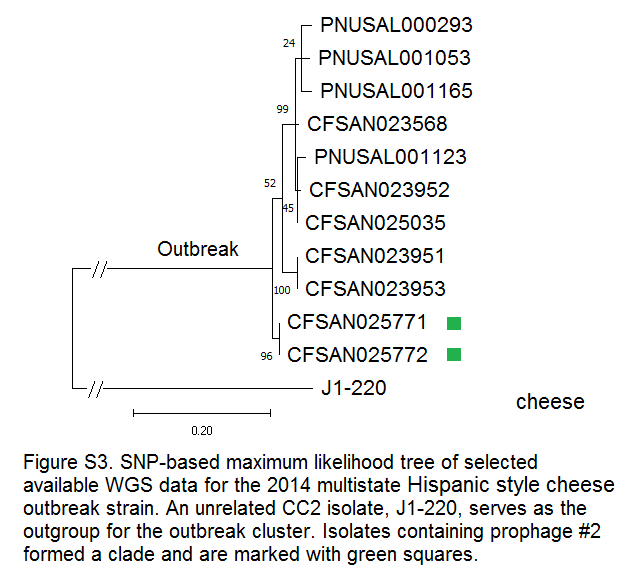

Supplement: Supplementary file 7 [file Image_3.tif]

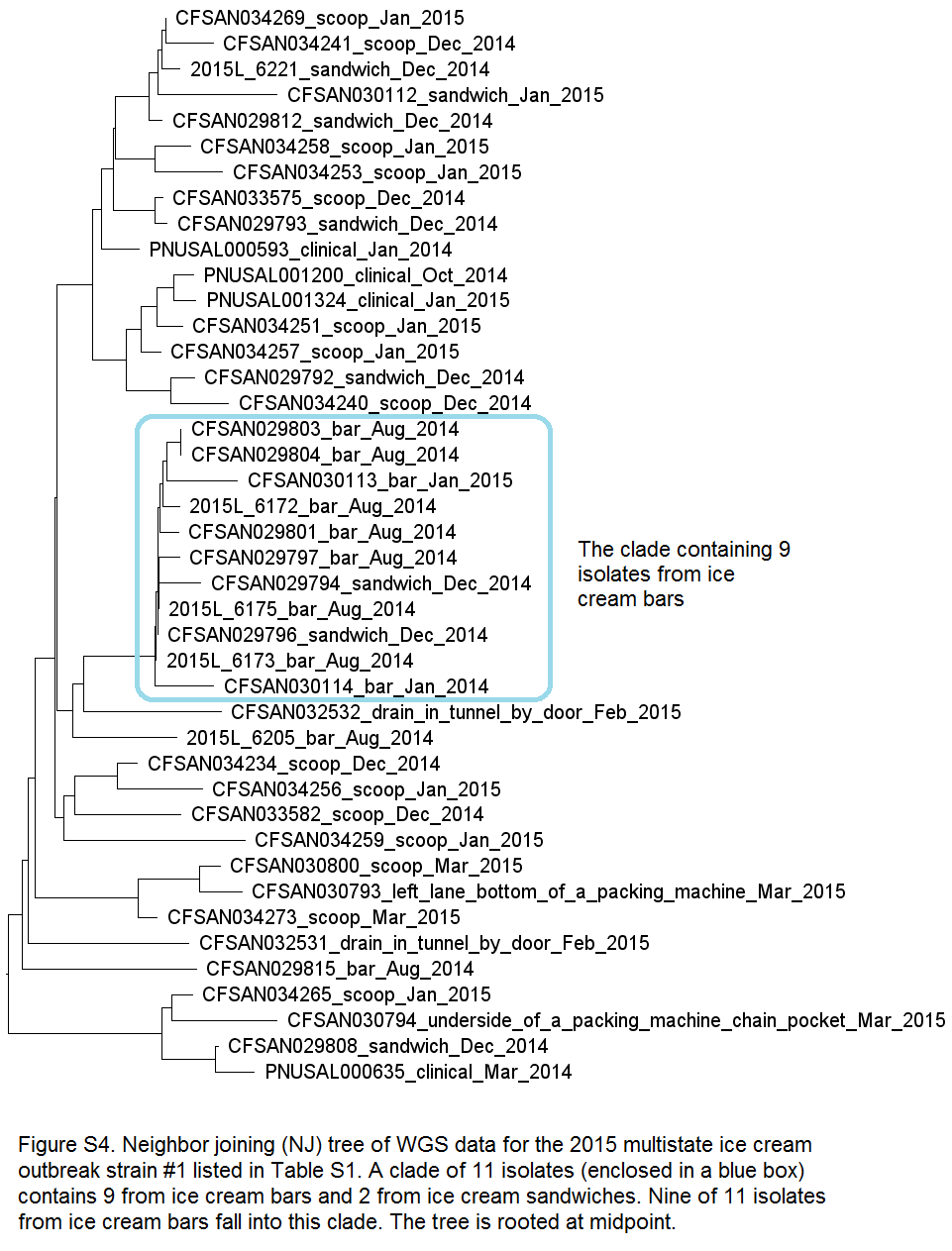

Supplement: Supplementary file 8 [file Image_4.tif]
